# Virome sequencing and analysis of *Aedes aegypti* and *Aedes albopictus* from ecologically different sites in the Philippines

**DOI:** 10.1186/s13071-025-07073-7

**Published:** 2025-10-24

**Authors:** Irish Coleen A. Asin, John Michael C. Egana, Richard E. Paul, Ma. Anita M. Bautista

**Affiliations:** 1 Functional Genomics Laboratory, National Institute of Molecular Biology and Biotechnology, University of the Philippines—Diliman, 1101 Quezon City, Philippines; 2 Institut Pasteur, CNRS UMR 2000, INRAE USC 1510, Ecology and Emergence of Arthropod-Borne Pathogens Unit, Université Paris Cité, 75015 Paris, France

**Keywords:** Virome, Metagenomics, Sequence-independent single-primer amplification, Insect-specific viruses, Land use change

## Abstract

**Background:**

*Aedes aegypti* and *Aedes albopictus* are important vectors of arthropod-borne viruses (arboviruses) such as dengue, chikungunya, and Zika. Changes in land use have long been considered a factor in the emergence of infectious diseases; thus, it is imperative to look at how the diversity of viruses is also affected by land use.

**Methods:**

Viral metagenomics was used to determine the virome compositions of 260 *Ae. aegypti* and 75 *Ae. albopictus* collected from the three study sites in Los Baños, Laguna, Philippines, that differ in topography and land use transformations.

**Results:**

The virome of *Ae. aegypti* and *Ae. albopictus* revealed virus sequences belonging to 12 different taxon groups, dominated by insect-specific viruses (ISVs) such as Phasi Charoen-like phasivirus (PCLV), Humaita Tubiacanga virus (HTV), and Wenzhou sobemo-like virus 4 (WSLV4). Both species were found to share the majority of identified viruses. Moreover, a relatively higher number of viral families were observed in sites that had undergone transformation from agriculture to bare and built-up areas, compared with a forest site.

**Conclusions:**

The findings of this study underscore the vast diversity of *Ae. aegypti* and *Ae. albopictus* viruses from the selected sites in the Philippines generated by viromics. Results also impact the understanding that land use may contribute to virus diversity. The prevalence of ISVs and nondetection of arboviruses in the virome composition of *Ae. aegypti* and *Ae. albopictus* were notable, suggesting further examination of the roles of ISVs in arbovirus transmission.

**Graphical abstract:**

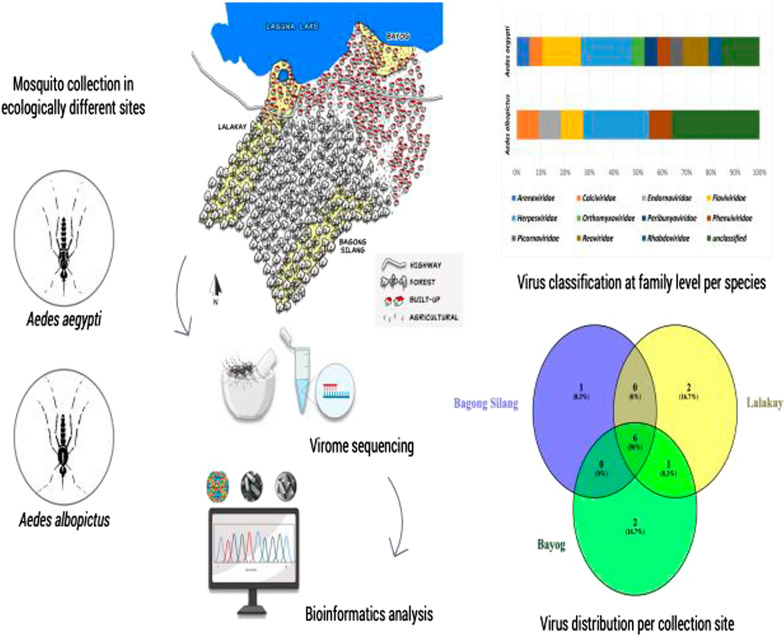

**Supplementary Information:**

The online version contains supplementary material available at 10.1186/s13071-025-07073-7.

## Background

Arboviruses including the mosquito-borne dengue virus (DENV), Zika virus (ZIKV), Japanese encephalitis virus (JEV), and chikungunya virus (CHIKV) are emerging and re-emerging worldwide. Global trade and travel, urbanization, and climate change are the main contributing factors to the continuous spread of these arboviruses that put about four billion people around the world at risk of mosquito-borne infection [1–4]. In the Philippines, a dengue epidemic was first recorded in 1954, and this disease has since remained endemic in all regions of the country [5, 6]. A record-breaking number of 437,563 dengue cases that resulted in 1689 deaths was reported nationwide in 2019, highlighting further the public health burden brought about by this disease [7]. The potential risk of some other, possibly unknown, emerging arboviral threat calls for strengthened surveillance programs and advanced research on arboviral diseases [8]. Arbovirus surveillance programs, which aim to regulate the emergence of new and old arboviral diseases by accurately monitoring the activity of endemic and emerging viruses in real time, are therefore vital public health priorities [9].

*Aedes aegypti* and *Ae. albopictus* are two of the most prevalent vectors of arboviruses. Often found in tropical and subtropical areas of the world, *Ae. aegypti* is the primary vector of arboviruses such as DENV, CHIKV, ZIKV, and yellow fever, among others [10]. In contrast, *Ae. albopictus* is most well-known for transmitting dengue and chikungunya viruses, but it has also been shown to be a competent vector for 22 other arboviruses [11, 12]. The occurrence of these two mosquito species is documented nationwide in the Philippines, rendering all regions of the country vulnerable to mosquito-borne infections [5, 13, 14]. Over the years, the interest in these two *Aedes* mosquitoes mainly lies in their importance as vectors of medically important pathogens. However, studies have shown that the mosquito virome—the collection of all mosquito-associated viruses, such as the dual-host arboviruses, insect-specific viruses, or bacteria- and fungi-infecting viruses—potentially impact the transmission of arboviruses [15–18]. In addition, sampling mosquitoes, known to feed on a myriad of hosts, can shed light on viral diversity over space, time, and species, and may serve as targets for zoonotic and arboviral disease surveillance programs [19, 20].

Land-use change (LUC) has been reported to be the major driver of emergence and reemergence of mosquito-borne diseases. Besides abolishing natural habitats and harming species, LUC is also affecting disease prevalence and distribution by creating novel niches for species living near humans, increasing breeding habitats and food resources, and altering vector–host relationships [21–23]. Furthermore, studies have shown that a diverse mosquito community composition varies significantly between forests and disturbed habitats and that the mosquito genera *Aedes, Anopheles,* and *Culex* were more commonly encountered in disturbed habitats and contained more virus isolates than forest mosquitoes [23, 24]. Indeed, LUC has a huge effect on local ecology and habitats, impacting mosquito abundance, species diversity, and, eventually, disease transmission.

In this study, the viromes of *Ae. aegypti* and *Ae. albopictus* collected from three sites in Los Baños, Laguna, Philippines, were explored. The three sites–Bagong Silang, Lalakay, and Bayog—differ ecologically, in terms of geographic elevation, topography, and land-use transformations. These three areas have documented records of anthropogenic modifications and have been the subject of assessment studies on the patterns and drivers of LUC [25]. Here, we present the first report on the viromes of Philippine-collected *Ae. aegypti* and *Ae. albopictus* from the three ecologically different sites, which may have affected the diversity of the viruses that the two *Aedes* mosquitoes harbor.

## Methods

### Mosquito collection, handling, storage, and identification

**Fig. 1:**
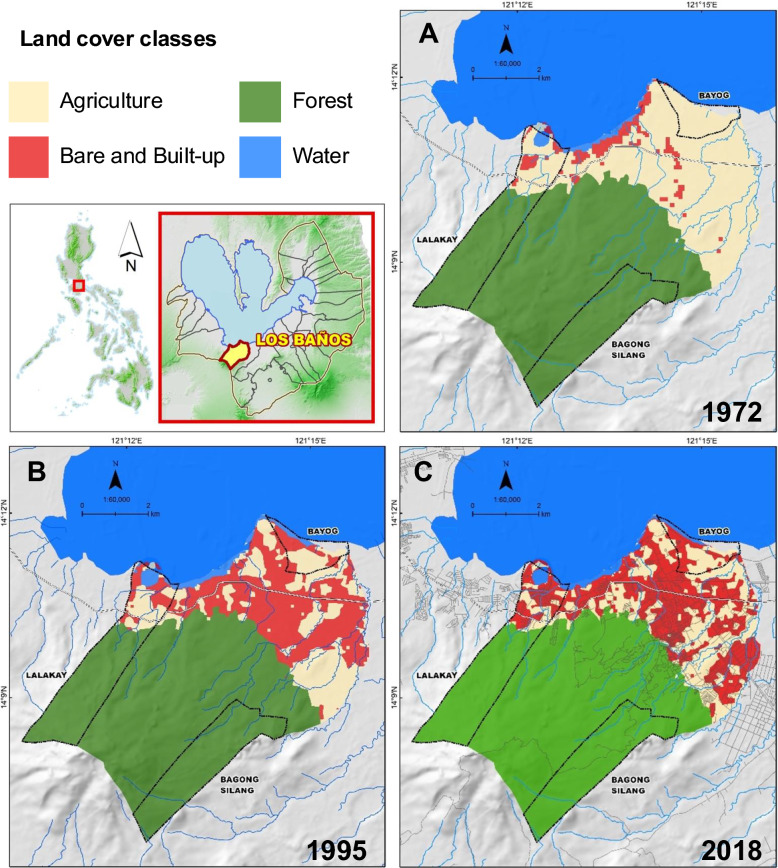
Land cover maps of Los Baños, Laguna, Philippines in (**A**) 1972, (**B**) 1995, and (**C**) 2018. Major land cover classes identified: forest, agriculture, and bare and built-up

*Aedes aegypti* and *Ae. albopictus* mosquito samples were collected during the months of April and May (hot, dry season), and August and October (rainy season) of 2018 from the three sites of Los Baños, Laguna, Philippines: Bagong Silang (14.13 N, 121.22 E), Lalakay (14.17 N, 121.21 E), and Bayog (14.192 N, 121.246 E). Land cover maps of Los Baños, Laguna (Fig. 1) depict the increase of built-up areas from 1972 to 1995 to 2018. Bagong Silang, Lalakay, and Bayog exhibit differences not only in geographic location but also in land cover classes observed. Bagong Silang is the mountain/upstream site with geographic elevation ranging from 305 to 331 m above sea level (asl). This study site is characterized by a rolling topography. Lalakay, on the other hand, is the midstream site and has a rolling to undulating topography. Bayog, the lakeshore/coastal site, is characterized by a flat topography. Based on the maps, Bagong Silang remained to have only forest cover throughout the span of 46 years, while Lalakay and Bayog both underwent conversion from agriculture to built-up areas during this period.

**Fig. 2:**
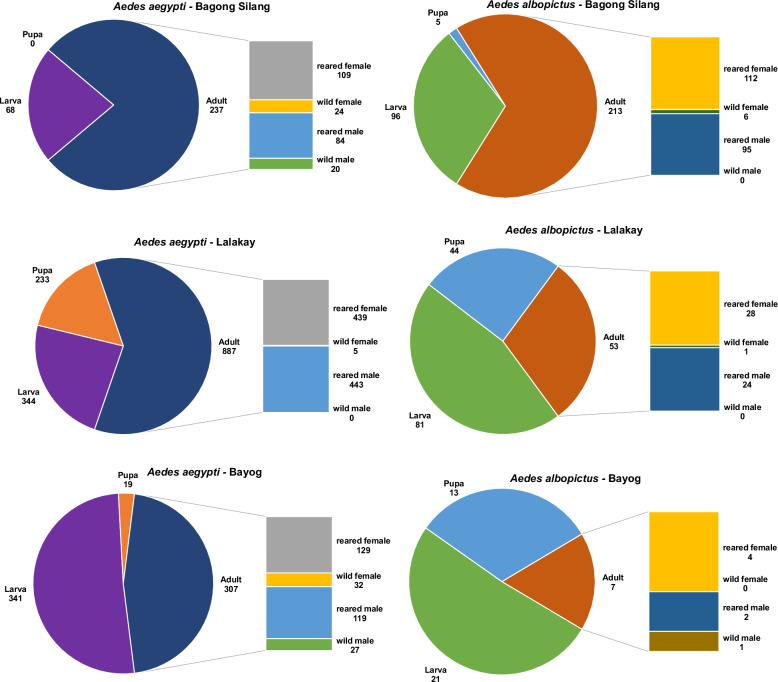
Distribution of collected *Aedes aegypti* and *Aedes albopictus* in terms of sex and developmental stage per site. Mosquitoes collected were sorted according to developmental stage: larva, pupa, and adult. Adult mosquitoes were further sorted according to sex (male or female) and whether they were reared to adult in the laboratory or caught as adult in the wild

Oviposition traps (ovitraps) and larval sampling for immature mosquitoes [see Additional File 1, Supplementary Text S1] and sweep nets for adults were used for the collection [14, 26]. Permission to sample was approved by the Makiling Center for Mountain Ecosystems (MCME) of the University of the Philippines Los Baños. A total number of 2,436 and 533 morphologically identified *Ae. aegypti* and *Ae. albopictus*, respectively, was recorded. Details of mosquito collection per site (Table 1) and per developmental stage, adult sexes, and whether they were wild-caught or reared to adult in the laboratory are summarized in Fig. 2. Mosquitoes—laboratory-emerged and field-collected—were stored in microcentrifuge tubes at −80 °C before use.

**Table 1:** Summary of *Aedes aegypti* (AE) and *Aedes albopictus* (ALB) collection

Species	Bagong Silang (305-331 m asl)			Lalakay (0-39 m asl)			Bayog (0-15 m asl)			Total
	Apr	Aug	Oct	May	Aug	Oct	May	Aug	Oct	
AE	90	90	125	1	1267	196	0	609	58	2,436
ALB	59	141	114	0	160	18	0	17	24	533

Collections were performed in April, August, and October 2018 in Bagong Silang, and in May, August, and October in Lalakay and Bayog. m asl, meters above sea level

Further identification of *Ae. aegypti* and *Ae. albopictus* was done on the basis of mitochondrial cytochrome c oxidase subunit I *(COI)* and the nuclear ribosomal internal transcribed spacer 2 *(ITS2)* amplification and sequencing. Primers for polymerase chain reaction (PCR), capillary sequencing results, and molecular phylogenetic analyses for both species are detailed in the Additional file 1: Supplementary Text S2, Table S1, Fig. S1, and Fig. S2.

### Virome sequencing and bioinformatics analysis

#### Mosquito sample set-ups

For virome sequencing, a total of 260 *Ae. aegypti* and 75 *Ae. albopictus* samples were pooled according to site, gender, and adult classification (Additional file 2: Supplementary Table S2). Except for the 30–50 ovitrap-collected and reared-to-adult *Ae. aegypti* samples from Lalakay, all other sample set-ups for sequencing used 15 individuals per pool to normalize the number of all adult mosquitoes from Lalakay, Bagong Silang, and Bayog. This was also done for consistency in the number of individuals pooled for a more accurate comparison of viral metagenomes between reared and field-caught *Ae. aegypti* adults. For *Ae. albopictus,* no set-up from Bayog was included owing to the low number (*n* = 7) of collected adults (reared or wild-caught) from the site. Wild-caught female and male samples were only included in *Ae. aegypti* set-ups collected from Bagong Silang and Bayog; no set-ups were processed for *Ae. aegypti* from Lalakay, as well as *Ae. albopictus* collected from all sites owing to the low number (*n* < 15) of wild-caught adults.

#### Sample preparation and viral RNA extraction

Two optimized sample preparation protocols [27, 28] for viral metagenomics were applied in this study. Pooled mosquitoes in sterile 1 × phosphate-buffered saline (PBS) were homogenized using sterile, ice-cold mortar and pestle. Homogenates were centrifuged at 13,000 × *g* for 30 min at 4 °C. Afterwards, approximately 1 mL of supernatant was collected, subjected to second centrifugation using the same conditions, and stored at –80 °C until use.

Supernatants (0.5×) from the previous step were subjected to pelleting by ultracentrifugation and brought up to 1 mL using 1 × PBS to fill ultracentrifuge tubes (Hitachi, Japan) to capacity. High-speed centrifugation was performed at 178,000 × *g* for 1 h at 10 °C using Hitachi Koki CPNX-100 ultracentrifuge (Hitachi, Japan). Pellets were resuspended in 150 μL of 1 × PBS and stored at –80 °C until further processing could be performed [28]. These samples were initially classified as “ultra-pure”; the other half volume that was not subjected to ultracentrifugation was labeled as “semi-pure.” Both set-ups were separately used as inputs for the succeeding steps, but no significant difference was eventually observed between them upon the completion of the whole virome analysis.

Prior to viral RNA extraction, 127 μL of supernatant (both semi- and ultra-pure samples) were treated with 14 units (U) of DNAse (Thermo Fisher Scientific, USA), 25U Benzonase® nuclease (Merck, Germany), and 20U Ambion™ RNase I (Thermo Fisher Scientific, USA) suspended in 10 × DNAse buffer (Thermo Fisher Scientific, USA). A total volume of 150 μL of nuclease-treated supernatant was then incubated at 37 °C for 1 h to digest unprotected nucleic acids. The nucleic acids packaged in viral capsids were then extracted using QIAamp Viral RNA Mini Kit (Qiagen, Germany) following the manufacturer’s protocol and stored at –80 °C until further use.

#### Reverse transcription and double-stranded cDNA (dscDNA) synthesis

A total of 10 µL of viral RNA were reverse transcribed using M-MLV Reverse Transcriptase (Thermo Fisher Scientific, USA) and specific anchored random primers (Additional file 2: Supplementary Table S3) as previously described [29]. To synthesize double-stranded cDNA (dscDNA), 2 µL of previously mentioned anchored random primers was added to 10 µL of single-stranded cDNA and incubated at 75 °C for 5 min and then on ice for 5 min for denaturation. Then, 1 µL of Klenow fragment (New England Biolabs, Singapore), 1 µL of 10 mM dNTPs (Thermo Fisher Scientific, USA), 2 µL of 10 × Klenow buffer (New England Biolabs, Singapore), and 6 µL of nuclease-free water (Vivantis Technologies, Malaysia) were added and the samples were incubated at 37 °C for 60 min, followed by 75 °C for 10 min. Afterwards, 0.5 µL of exonuclease I (New England Biolabs, Singapore), 1 µL of shrimp alkaline phosphatase (New England Biolabs, Singapore), 5 µL of 10 × phosphatase buffer (New England Biolabs, Singapore), and 22 µL of DEPC-treated H_2_O (Thermo Fisher Scientific, USA) were incorporated to the mixture and incubated at 37 °C for 60 min followed by 75 °C for 10 min to eliminate the phosphates and the free single-strand nucleic acid in the dscDNA reaction.

#### Sequence-independent single-primer amplification (SISPA)

Double-stranded cDNA (dscDNA) was prepared in a 50 µL-reaction containing 10 µL of dscDNA, 3 µL of barcode primer (Additional file 2: Supplementary Table S3), 0.5 µL of PrimeSTAR HS DNA polymerase (Takara Bio Inc., USA), 10 µL of 5 × PrimeSTAR buffer (Takara Bio Inc., USA), 4 µL of dNTP mixture (Takara Bio Inc., USA), and 22.5 µL nuclease-free water (Vivantis Technologies, Malaysia). The PCR condition used was set at 95 °C for 3 min; 40 cycles of 95 °C for 20 s, 54 °C for 20 s, and 68 °C for 70 s; and a final extension at 68 °C for 7 min. SISPA products were purified using Agencourt AMPure XP beads (Beckman Coulter Inc., USA) following a 1.8X AMPure XP beads to SISPA products volume ratio to remove fragments < 100 bp.

#### Illumina MiSeq sequencing

Library preparation using ~500 ng input of purified SISPA products was performed following Illumina’s Nextera™ DNA Flex Library Prep Reference Guide and workflow. Libraries were evaluated using the Agilent 4200 TapeStation (Agilent, USA). Quantitative real-time polymerase chain reaction (RT-qPCR) using the Kapa Library Quantification Kit (Kapa Biosystems, USA) was also performed. The pooled library was then diluted to a final loading concentration of 12 pM following the MiSeq System Denature and Dilute Libraries Guide (Illumina Technologies, USA). A 5% spike-in of PhiX control library was also used [30]. The denatured and diluted libraries were loaded in Illumina MiSeq (Illumina Technologies, USA) for 2 × 250 bp paired-end sequencing.

#### Bioinformatics analysis, validation of sequences, and phylogenetic analysis

**Fig. 3:**
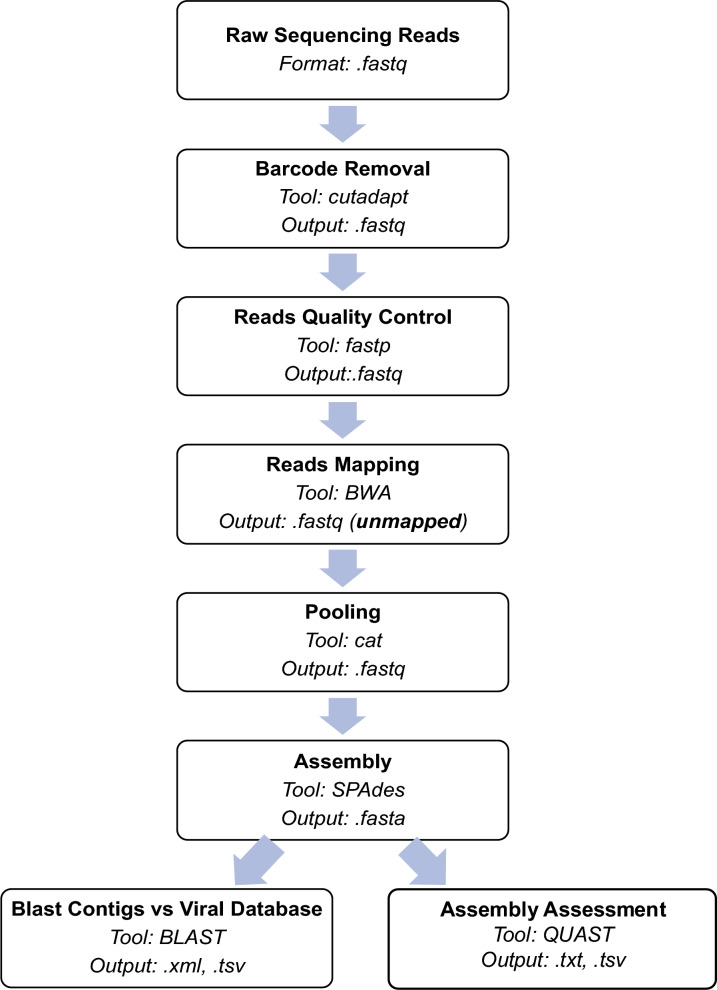
Bioinformatics workflow for virome analysis of *Aedes aegypti* and *Aedes albopictus* samples. Cutadapt was used to remove barcodes that were added to the samples prior library preparation and quality filtering of reads was done using fastp. Burrows-Wheeler Aligner (BWA) was then used to remove reads that aligned to mosquito host reference genomes (*Ae. aegypti*: GCF_002204515.2; *Ae. albopictus*: GCF_001876365.2). Sequencing reads of replicate samples from laboratory-emerged adults were pooled using cat, followed by *de novo* assembly using SPAdes. QUAST (Quality Assessment Tool) was then used to assess the quality of assemblies and all the assembled contigs were queried against the Reference Viral Database (RVDB16.0) using BLAST. Viral sequences with significant BLAST hits (E value < 0.00001) were summarized and analyzed. A more in-depth comparative analysis of top BLAST hit virus sequences with aligned length at least 100 bp was performed

Virome analysis was developed and performed as depicted in Fig. 3 with the assistance of the Core Facility for Bioinformatics of the Philippine Genome Center (CFB-PGC). To validate the viruses found, primers were designed using PrimerBLAST [31] to target specific virus sequences (i.e., polyprotein, RdRP, glycoprotein precursor, nucleocapsid, hypothetical proteins) of the longest contigs (bp) that were *in silico* detected. Primers for these viruses are presented in Additional file 2: Supplementary Table S4. PCR was performed using Invitrogen 2 × PCR Master Mix (Thermo Fisher Scientific, USA) and the cycling conditions used include an initial denaturation of 5 min at 95 °C, followed by 35 cycles of 30-s denaturation at 95 °C, 30-s annealing at 55 °C and 1-min extension at 72 °C, and a final extension for 5 min at 72 °C. Amplicons with expected sizes were purified using either Agencourt AMPure XP (Beckman Coulter, USA) or Freeze 'N Squeeze™ DNA Gel Extraction Spin Columns (Bio-Rad Laboratories, Inc., USA) following the manufacturers’ instructions, and then submitted to the Philippine Genome Center for capillary sequencing. The quality of the sequences was assessed, and the identities of viruses were cross-referenced in Refence Viral Database (RVDBv16.0; same version used in bioinformatics analysis) using BLAST.

To assess the evolutionary relationship of the viruses in this study with other known viruses, phylogenetic analysis was done on the basis of the gene-coding region of the virus sequences and related sequences deposited in GenBank. Gene-coding regions analyzed were flavivirus polyprotein gene (NS4B product) of cell-fusing agent virus (CFAV), hypothetical protein 1 of Hubei mosquito virus 2 (HMV2), capsid of Humaita-Tubiacanga virus (HTV), RNA-dependent RNA polymerases of Merida virus (MERDV) and Phasi Charoen-like phasivirus (PCLV), and hypothetical protein 2 of Wenzhou sobemo-like virus 4 (WSLV4). Multiple sequence alignment and phylogenetic tree building were done using MEGA 7 [32]. Alignment of the virus sequences in the study and closely related sequences (5–10 sequences if available in GenBank) based on BLAST results was performed using MUSCLE. The best DNA model for evolutionary phylogenetic inference was then chosen on the basis of the lowest Bayesian Information Criterion (BIC) scores. Phylogenetic trees were then computed using the Maximum Likelihood method with 1000 bootstrap replicates.

## Results

### Virome sequencing and bioinformatics analysis

#### *Sequencing reads assessment, *de novo* assembly, and annotation*

Raw read counts per sample and read counts retained after every step of the bioinformatics analysis prior *de novo* assembly for *Ae. aegypti* and *Ae. albopictus* samples are presented in Table 2**; **Additional file 2: Supplementary Table S5. A total of 5,132,635 and 1,798,188 raw reads were obtained from *Ae. aegypti* and *Ae. albopictus* samples, respectively. Quality filtering reduced read counts to 74.33% of the total raw reads for *Ae. aegypti* and 59.41% for *Ae. albopictus* samples. Cleaned reads were mapped to mosquito host reference sequences and the unmapped reads accounted for 72.98% and 62.79% of the cleaned reads from *Ae. aegypti* and *Ae. albopictus* samples, respectively. Results suggest that despite the use of nucleases to remove host nucleic acids during mosquito sample preparation, approximately one third of the reads were contaminating RNAs from host mosquitoes.

**Table 2:** Read counts and contigs obtained from *Aedes aegypti* and *Aedes albopictus* samples

Result	AE-BS	AE-L	AE-B	Total	ALB-BS	ALB-L	Total
Total no. of samples	60	140	60	260	45	30	75
Raw reads	1,219,116	2,020,666	1,892,853	5,132,635	962,814	835,374	1,798,188
Cleaned reads	793,234	1,880,353	1,141,705	3,815,292	527,509	540,792	1,068,301
% Cleaned	65.07	93.06	60.32	74.33	54.79	64.74	59.41
Filtered reads	692,438	1,205,709	886,186	2,784,333	437,554	233,203	670,757
% Filtered	87.29	64.12	77.62	72.98	82.95	43.12	62.79
Assembled contigs	17,329	68,777	16,122	102,228	22,701	6,433	29,134
Classified as viruses	3,935	20,727	2,960	27,622	700	264	964

Raw, cleaned (> Q15), and filtered (removal of mosquito host) clean reads are tallied per species and collection site. Percentage of reads retained are also detailed after cleaning and unmapping of reads. Assembled contigs and contigs with viral hits are also included. Mosquito sample: AE, *Aedes aegypti*; ALB, *Aedes albopictus.* Collection sites: BS, Bagong Silang; L, Lalakay; B, Bayog

*De novo* assembly yielded 102,228 and 29,134 assembled contigs for *Ae. aegypti* and *Ae. albopictus.* Of the total number of contigs identified, 18.7% (19,118) were longer than 100 bp for *Ae. aegypti* and 15.35% (4,472) for *Ae. albopictus* (Additional file 2: Supplementary Table S6). The average N50 for *Ae. aegypti* was 265 with 4097 bp as the largest contig assembled. For *Ae. albopictus,* the average N50 was 145 and 2603 bp was the largest contig assembled. After querying the assembled contigs against RVDB using BLAST, the total number of contigs with matched virus sequences for *Ae. aegypti* was 27,622 contigs (27.02% of the total assembled contigs). For *Ae. albopictus*, only 964 contigs (3.31% of the total contigs assembled) matched with virus sequences (Table 2).

Assembly and annotation statistics showed a quarter of the total assembled contigs with significant BLAST hits to virus databases for *Ae. aegypti*. A smaller proportion (< 5%) was observed for *Ae. albopictus.* The RVDB version used in this study was accessed last 2019 and the database was not as populated with virus sequences compared with the latest version. With several new sequences deposited in the database since then, use of a more recent viral database could potentially increase the percentage of contigs with matched virus sequences.

#### Virus classification on the basis of host type and family

**Fig. 4:**
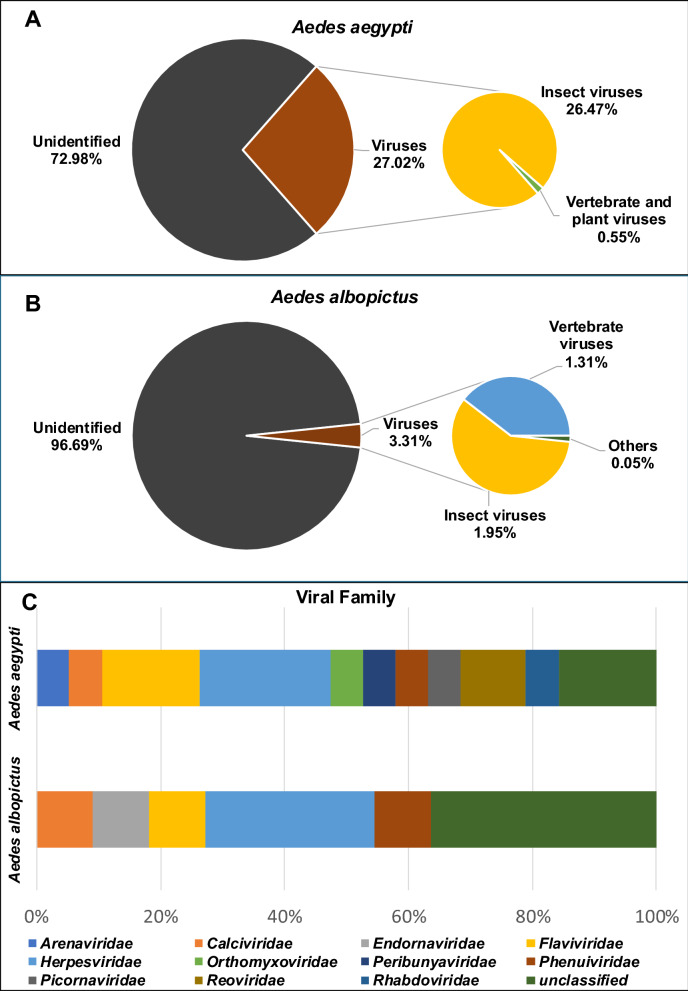
Classification of *Aedes aegypti* and *Aedes albopictus* viromes on the basis of host and viral families. Viruses identified from *Ae. aegypti* (**A**) and *Ae. albopictus* (**B**) were further classified by host types and according to viral families (**C**). Classification was based on the total number of assembled contigs

The majority of the identified viruses based on all the assembled contigs from *Ae. aegypti* were insect viruses (26.47%), which were dominated by mosquito-specific viruses **(**Fig. 4A**)**. For *Ae. albopictus,* a much lower percentage (only 3.31%) accounted for virus-like contigs. Aside from insect viruses (1.95%), vertebrate viruses (1.31%) comprise most of the annotated viruses **(**Fig. 4B**)**, and other viruses from plants, other invertebrates, and environmental samples constituted the minority (< 1%) for both species (Additional file 3: Supplementary Table S7, Table S8).

**Fig. 5:**
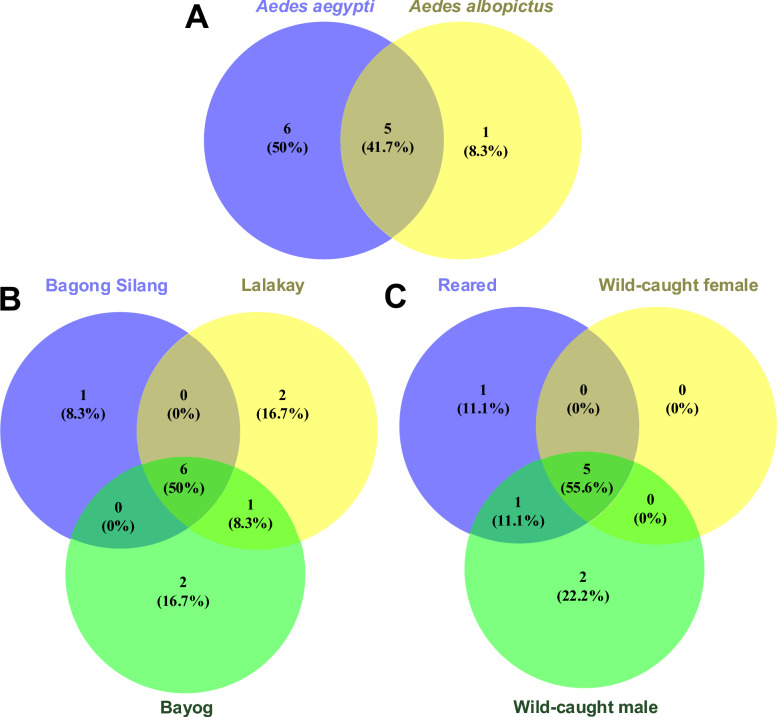
Venn diagram of viral families sorted according to host species, collection site, and adult type. Viral families were distributed according to (**A**) mosquito host (*Aedes aegypti* and *Aedes albopictus*), (**B**) collection site (Bagong Silang, Lalakay, and Bayog) regardless of mosquito species, and (**C**) type of *Aedes aegypti* adult (reared/laboratory emerged, or wild-caught male and female adults) collected in Bagong Silang and Bayog

Based on taxonomic classification at the family level, a total of 12 taxon groups were identified from *Ae. aegypti* and *Ae. albopictus* top hit viral contigs (Fig. 4C and Fig. 5A)*. Calciviridae, Flaviviridae, Herpesviridae, Phenuiviridae*, and a group of unclassified viruses were shared by the two species. Meanwhile, *Arenaviridae, Orthomyxoviridae, Peribunyaviridae, Picornaviridae, Reoviridae,* and *Rhabdoviridae* were exclusively found in *Ae. aegypti* samples; *Endornaviridae* was only found in *Ae. albopictus* samples (Additional file 3: Supplementary Table S9).

#### Virus distribution among collection sites and adult type

Viral families were further compared according to the collection site (Bagong Silang, Lalakay, and Bayog) as seen in Fig. 5B. In *Ae. aegypti*, six families (*Calciviridae, Flaviviridae, Herpesviridae, Phenuiviridae, Reoviridae*, and unclassified viruses) were shared across all sites whereas *Picornaviridae* was common between Lalakay and Bayog. While *Endornaviridae* was solely identified in *Ae. albopictus* samples from Bagong Silang, *Orthomyxoviridae* and *Peribunyaviridae* were exclusively found in Lalakay, and *Arenaviridae* and *Rhabdoviridae* in Bayog for *Ae. aegypti* samples. Interestingly, no viral families were shared between Bagong Silang and Lalakay, nor between Bagong Silang and Bayog.

Viruses were also classified according to adult host type: 1) laboratory-reared adults; 2) wild/field-caught female adults; and 3) wild/field-caught male adults (Fig. 5C). *Calciviridae, Flaviviridae, Herpesviridae, Phenuiviridae,* and unclassified viruses were found to be common among the three groups, while *Reoviridae* was shared between reared adults and wild-caught males. *Arenaviridae* was exclusively identified in reared adults, whereas *Picornaviridae* and *Rhabdoviridae* were only detected in wild males. No viral family was solely identified in wild-caught females.

#### Virus composition at the species level

To further analyze the virome of *Ae. aegypti* and *Ae. albopictus* at the species level, contigs with at least 100-bp aligned length to virus sequences were retained [29]. This resulted in only seven viral species identified from *Ae. aegypti* and six from *Ae. albopictus* (Table 3). Information on each virus, such as viral family, segment type, region of alignment, no. of contigs, size range of aligned length (bp), percent identity, and percent query cover were detailed. Excluding bacteria-like contigs and uncultured/environmental sample sequences, the viral species are discussed hereinafter.

**Table 3:** Viruses from *Aedes aegypti* and *Aedes albopictus* samples

Virus, Family	Classification	AE-BS	AE-L	AE-B	ALB-BS	ALB-L
Cell-fusing agent virus (CFAV),	Presence ( +) or absence (-)	–	+	−	−	−
*Flaviviridae*	Region of alignment		polyprotein			
	No. of contigs ≥ 70 bp		14			
	Contig size range (bp)		106–725			
	Identity (%)		95.9–98.2			
	Query cover (%)		44.5–100			
Humaita-Tubiacanga virus (HTV), unassigned	Presence (+) or absence (−)	** + **	** + **	** + **	** + **	
	Region of alignment	capsid, RdRP	capsid, RdRP	capsid, RdRP	RdRP	
	No. of contigs ≥ 70 bp	58	977	81	2	
	Contig size range (bp)	90–2685	70–685	73–2224	467–526	
	Identity (%)	95.8–100	88.1–100	91.6–100	92.4–97.9	
	Query cover (%)	47.5–100	32.3–100	42.1–100	79.7–100	
Merida virus (MERDV),	Presence (+) or absence (−)	−	−	**+**	**–**	−
*Rhabdoviridae*	Region of alignment			nucleoprotein, RdRP		
	No. of contigs ≥ 70 bp			6		
	Contig size range (bp)			307–893		
	Identity (%)			95.2–97.2		
	Query cover (%)			93.6–100		
Phasi Charoen-like phasivirus (PCLV), *Phenuiviridae*	Presence (+) or absence (−)	+	+	+	+	+
	Region of alignment	L, M, S segments	L, M, S segments	L, M, S segments	L, M, S segments	L and M segments
	No. of contigs ≥ 70 bp	521	983	189	3	4
	Contig size range (bp)	71–3243	70–706	70–4097	357–394	236–342
	Identity (%)	85.7–100	87.9–100	91.7–100	93.3 -96.5	88.6–96.8
	Query cover (%)	28.6–100	36.7–100	31.4–100	99.2–100	60.4–97.7
Whidbey virus (WHIDV),	Presence ( +) or absence (−)	−	+	−	−	−
*Orthomyxoviridae*	Region of alignment		PB2 gene			
	No. of contigs ≥ 70 bp		1			
	Contig size range (bp)		192			
	Identity (%)		79.69			
	Query cover (%)		57.58			
Hubei mosquito virus 2	Presence (+) or absence (-)	−	−	−	−	+
(HMV2), unclassified	Region of alignment					hypothetical protein 1
	No. of contigs ≥ 70 bp					2
	Contig size range (bp)					74–464
	Identity (%)					74.4–93.2
	Query cover (%)					29.7–43.3
Wenzhou sobemo-like virus 4	Presence (+) or absence (−)	+	−	−	−	+
(WSLV4), unclassified	Region of alignment	hypothetical protein 1 and 2				hypothetical protein 1 and 2
	No. of contigs ≥ 70 bp	3				12
	Contig size range (bp)	1396–1457				71–2535
	Identity (%)	77.9–78.2				93–99
	Query cover (%)	50.2–62.2				61.6–100

The following information is included per virus: segment type, virus family, matched gene region, number of contigs, sizes of contigs, percent identity, and query cover. Presence and absence per site are denoted by + and −, respectively. Species: AE, *Aedes aegypti*; ALB, *Aedes albopictus*; Collection sites: BS, Bagong Silang; L, Lalakay; B, Bayog

### Positive-sense single-stranded RNA viruses (+ ssRNA) viruses

Two (2) positive-sense (+), single-stranded (ssRNA) viruses were detected: the cell-fusing agent virus (CFAV) and the Humaita-Tubiacanga virus (HTV). The cell-fusing agent virus belongs to *Flaviviridae.* The longest assembled CFAV-like contig (632 bp) has 96.36% nucleotide (nt) sequence identity to CFAV (NCBI Accession no.: NC_001564.2) from *Ae. aegypti* collected in the USA in 2012. The HTV, on the other hand, is an unclassified (+) ssRNA. The longest HTV-like contigs from *Ae. aegypti* samples (2717 bp and 1508 bp) have 97.39% and 97.88% nucleotide sequence similarity to HTV RdRP (KR003801.1) and HTV capsid (KR003802.1) from Brazil, respectively. A relatively shorter HTV-like contig (526 bp), on the other hand, was detected from *Ae. albopictus* which has 92.4% nt identity to HTV RdRP from Brazil.

### Negative-sense single-stranded RNA viruses (– ssRNA) viruses)

Next to these are three viruses that are negative-sense (–), ssRNA viruses: Merida virus (MERDV), Phasi Charoen-like phasivirus (PCLV), and Whidbey virus (WHIDV). The Merida virus (MERDV) is from *Rhabdoviridae.* The longest MERDV-like contig (893 bp) exhibited 95.74% nucleotide identity to MERDV RdRP (MH310083) from *Cx. quinquefasciatus* collected from the USA in 2015. On the other hand, PCLV is from *Phenuiviridae.* In *Ae. aegypti,* the longest contig (4097 bp) aligned to PCLV has 97.88% nt identity to PCLV RdRP from Thailand (KM001085; 6783 bp). High nucleotide percent identities were similarly observed in the longest contig assembled that matched the glycoprotein gene (96.61%; 2829 bp) and nucleocapsid gene (96.62%; 1360 bp) of the virus. PCLV glycoprotein-like longest contig was most similar to PCLV glycoprotein from Brazil (NC_038261.1, 3935 bp), whereas the PCLV nucleocapsid-like longest contig was similar to PCLV nucleocapsid from Thailand (KM001087, 1389 bp). In *Ae. albopictus,* the longest viral contigs exhibit high nucleotide identities to PCLV RdRP (96.45%) and PCLV glycoprotein (95.38%) from Thailand, and to PCLV nucleocapsid isolate Rio from Brazil and strain Aag2-Bristol from the UK (93.28%). However, comparing the length of contigs with those from *Ae. aegypti*, the sizes of the longest contig that matched to PCLV RdRP, glycoprotein, and nucleocapsid were significantly shorter (394 bp, 390 bp, and 357 bp, respectively). Whidbey virus (WHIDV) of *Orthomyxoviridae* was also present, but with only a 192-bp aligned contig length (79.69% similarity) to Whidbey virus strain UW1 (KX898492.1) detected from *Aedes dorsalis* in the USA.

### Unclassified RNA viruses

Two unclassified RNA viruses, Hubei mosquito virus 2 (HMV2) and Wenzhou sobemo-like virus 4 (WSLV4), were also identified. The HMV2 was solely identified in *Ae. albopictus* samples. The longest contig of HMV2 is 464 bp and shows 74.35% nucleotide sequence identity to HMV2 from China (NC_033306.1; 1652 bp). Meanwhile, WSLV4 was detected from both *Aedes* species, and both are most similar to WSLV4 sequences (NC_033138.1) from China. However, higher nucleotide identity (96.96%) was observed on the longest WSLV4-like contig (2535 bp) from *Ae. albopictus* compared with the longest WSLV4-like contig (1457 bp) from *Ae. aegypti* (78.17%).

### Validation and phylogeny of detected virus sequences

The accuracy of the resulting assembly of viral contigs in this study was assessed using polymerase chain reaction (PCR) with at least two primer pairs designed to target each virus that was *in silico*-detected from both *Ae. aegypti* and *Ae. albopictus* samples. Capillary sequencing and BLAST results are summarized in Additional file 3: Supplementary Table S10, Table S11 (for *Ae. aegypti*), and Supplementary Table S12 (for *Ae. albopictus*). Phylogenetic trees of the six validated viruses CFAV, HMV2, HTV, MERDV, PCLV, and WSLV4 were also constructed.

#### Positive-sense single stranded RNA viruses

**Fig. 6:**
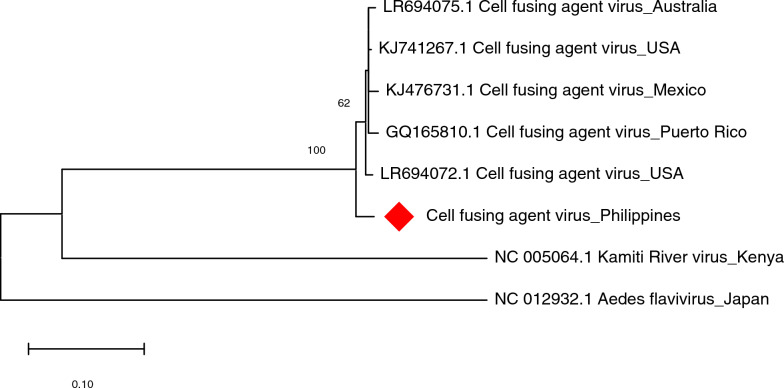
Molecular phylogenetic analysis of cell-fusing agent virus (CFAV) of *Aedes aegypti*. The evolutionary history was inferred by using the Maximum Likelihood method based on the Kimura 2-parameter model. Analysis of the flavivirus polyprotein gene of CFAV involved eight nucleotide sequences and a total of 350 positions in the final dataset. Values on the nodes represent bootstrap values based on 1000 bootstrap replicates; bootstrap values below 50% are not shown. Evolutionary analyses were conducted in MEGA7. The CFAV sequence in this study is labeled with a red rhombus

The *in silico*-detected CFAV from *Ae. aegypti* samples, was also evident in PCR results of Lalakay and Bayog sample set-ups using CFAV-P1 (~482 bp) and CFAV-P2 (~408 bp) primer sets. Capillary sequencing and BLAST of all samples indicated 95–99% sequence identity with CFAV polyprotein gene sequences from the USA and Puerto Rico, as detailed in Additional file 3: Supplementary Table S13. Using the longest CFAV-like contig detected (see Additional file 3: Supplementary Table S14), the phylogeny of CFAV was constructed as shown in Fig. 6. Results showed clustering with CFAV sequences from the USA, Australia, Mexico, and Puerto Rico with 100% nodal support. Inclusion of CFAV sequences from Thailand and Cambodia resulted in low nodal support (< 70%). Notably, a previous study revealed discrepancies between geography and phylogeny of CFAV, specifically in Southeast Asia, where CFAV sequences from Cambodia and Thailand diverged significantly [33].

HTV was found to be present in *Ae. aegypti* collected from the three sites, and in *Ae. albopictus* from Bagong Silang based on the results of bioinformatics analysis. PCR results, however, showed no amplification using primer set HTV-R (targets RdRP), but successful amplifications (~435 bp) for all *Ae. aegypti* samples using the primer set HTV-C that targets the capsid gene of the virus. A similarity of 94–99% with HTV capsid gene sequences in Brazil was observed. Using both primer sets (HTV-R and -C), there was no amplification produced for *Ae. albopictus* samples.

**Fig. 7:**
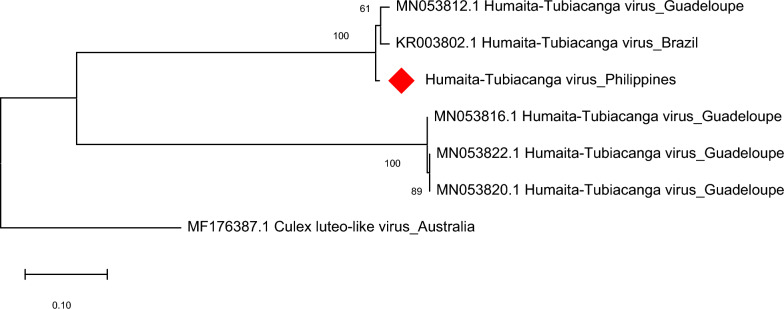
Molecular phylogenetic analysis of Humaita-Tubiacanga virus (HTV) of *Aedes aegypti*. The evolutionary history was inferred by using the Maximum Likelihood method based on the Tamura 3-parameter model. Analysis of the capsid gene involved seven nucleotide sequences and a total of 607 positions in the final dataset. Values on the nodes represent bootstrap values based on 1000 bootstrap replicates; bootstrap values below 50% are not shown. Evolutionary analyses were conducted in MEGA7. The HTV sequence in this study is labeled with a red rhombus

**Fig. 8:**
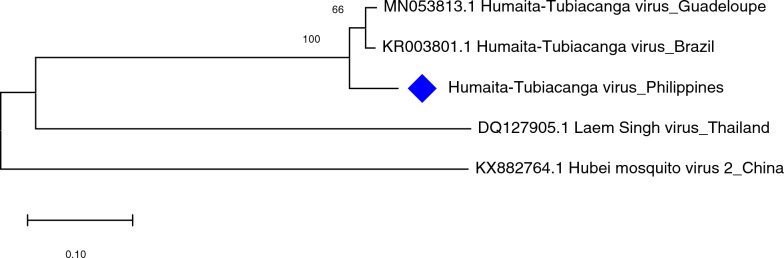
Molecular phylogenetic analysis of Humaita-Tubiacanga virus (HTV) of *Aedes albopictus*. The evolutionary history was inferred by using the Maximum Likelihood method based on the Kimura 2-parameter model. Analysis of the RNA-dependent RNA polymerase (RdRP) gene involved five nucleotide sequences and a total of 377 positions in the final dataset. Values on the nodes represent bootstrap values based on 1000 bootstrap replicates; bootstrap values below 50% are not shown. Evolutionary analyses were conducted in MEGA7. The HTV sequence in this study is labeled with a blue rhombus

The HTV of *Ae. aegypti* from Bagong Silang showed a close relationship as well (100% nodal support) with HTV sequences in Brazil and Guadeloupe based on evolutionary analysis of the capsid gene of the virus (Fig. 7**)**. Analysis of HTV RdRP produced similar clustering results in *Ae. albopictus* (Fig. 8).

#### Negative-sense single-stranded RNA

**Fig. 9:**
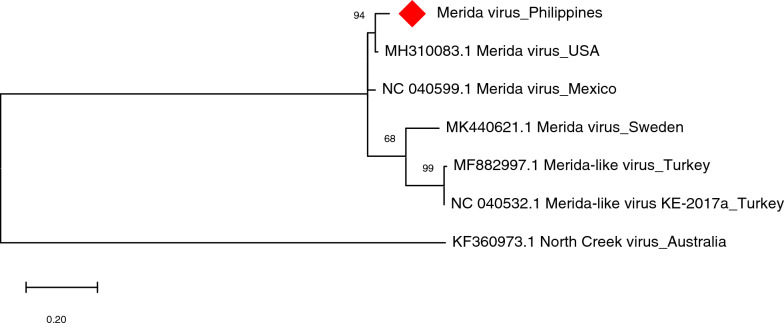
Molecular phylogenetic analysis of Merida virus (MERDV) of *Aedes aegypti*. The evolutionary history was inferred by using the Maximum Likelihood method based on the Kimura 2-parameter model. Analysis of the RNA-dependent RNA polymerase (RdRP) gene involved seven nucleotide sequences and a total of 287 positions in the final dataset. Values on the nodes represent bootstrap values based on 1000 bootstrap replicates; bootstrap values below 50% are not shown. Evolutionary analyses were conducted in MEGA7. The MERDV sequence in this study is labeled with a red rhombus

The MERDV detected *in silico* in male adult *Ae. aegypti* collected from Bayog, was successfully amplified using primer set MERDV-R (~585 bp). BLAST results indicate 90–96% similarity with MERDV sequences found in the USA and Mexico. This result is further supported by the maximum likelihood tree of MERDV that shows close relationships with MERDV from the USA and Mexico (Fig. 9).

**Fig. 10:**
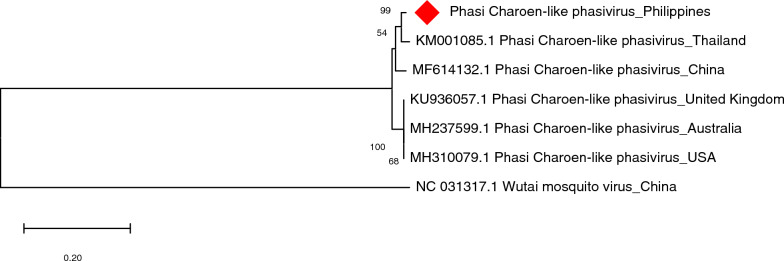
Molecular phylogenetic analysis of Phasi Charoen-like phasivirus (PCLV) of *Aedes aegypti*. The evolutionary history was inferred by using the Maximum Likelihood method based on the Hasegawa-Kishino-Yano model. Analysis of the RNA-dependent RNA polymerase (RdRP) gene involved seven nucleotide sequences and a total of 933 positions in the final dataset. Values on the nodes represent bootstrap values based on 1000 bootstrap replicates; bootstrap values below 50% are not shown. Evolutionary analyses were conducted in MEGA7. The PCLV sequence used in this study is labeled with a red rhombus

**Fig. 11:**
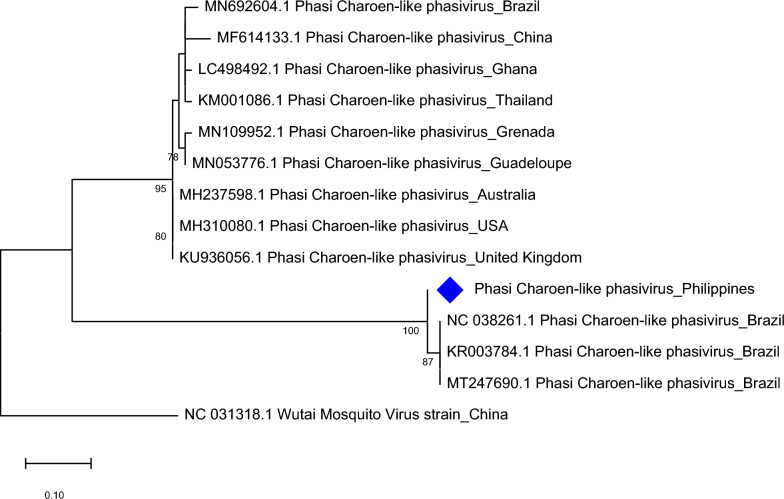
Molecular phylogenetic analysis of Phasi Charoen-like phasivirus (PCLV) of *Aedes albopictus*. The evolutionary history was inferred by using the Maximum Likelihood method based on the Tamura 3-parameter model. Analysis of the glycoprotein precursor involved 14 nucleotide sequences and a total of 106 positions in the final dataset. Values on the nodes represent bootstrap values based on 1000 bootstrap replicates; bootstrap values below 50% are not shown. Evolutionary analyses were conducted in MEGA7. The PCLV sequence used in this study is labeled with a blue rhombus

PCLV was *in silico*-detected in both *Ae. aegypti* and *Ae. albopictus*. PCR results from *Ae. aegypti* samples collected from all sites showed successful amplifications of the L, M, and S segments of PCLV. Results of capillary sequencing and BLAST showed that PCLV L segment from most of the *Ae. aegypti* samples were found to be 94–98% similar to PCLV sequences from Thailand and Brazil; some resulted in lower percent identity (< 90%) but with similar geolocation. Meanwhile, PCLV M segment exhibited 94–98% similarity with sequences from Australia and Thailand and PCLV S segment showed 93–98% sequence similarity with those from Thailand, Brazil, China, USA, and the UK. The maximum likelihood tree of PCLV from *Ae. aegypti* collected in Bayog revealed a close relationship (99% nodal support) with PCLV from Thailand. Analysis was done on the partial RdRP sequence of the virus, as shown in Fig. 10. PCLV was also amplified in *Ae. albopictus samples* using primers for L and M segments. After sequencing and querying using BLAST, PCLV segment L was found to be 96–97% similar to sequences from China, Thailand, USA, and Brazil. On the other hand, PCLV segment M or glycoprotein precursor exhibited 97% sequence similarity with PCLV from Thailand. For the phylogeny, the tree was constructed on the basis of the M segment. As observed in Fig. 11**,** a distinct clade of PCLV from Brazil and this study was formed with 100% nodal support.

#### Unclassified RNA viruses

**Fig. 12:**
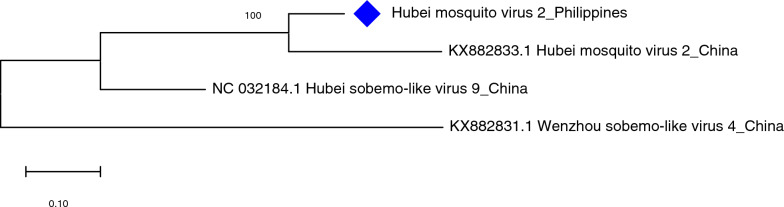
Molecular phylogenetic analysis of Hubei mosquito virus 2 (HMV2) of *Aedes albopictus.* The evolutionary history was inferred by using the Maximum Likelihood method based on the Kimura 2-parameter model. Analysis of the hypothetical protein 1 of HMV2 involved four nucleotide sequences and a total of 414 positions in the final dataset. Values on the nodes represent bootstrap values based on 1000 bootstrap replicates; bootstrap values below 50% are not shown. Evolutionary analyses were conducted in MEGA7. The HMV2 sequence used in this study is labeled with a blue rhombus

HMV2 was exclusively identified in *Ae. albopictus* samples on the basis of *in silico* analysis. PCR- and sequencing-validation showed only 71–74% similarity with HMV2 sequences from China, which may be indicative of HMV2-like variants found in *Ae. albopictus* samples. The phylogenetic tree, however, showed a close relationship with HMV2 sequence from China with 100% maximum likelihood bootstrap values (Fig. 12).

**Fig. 13:**
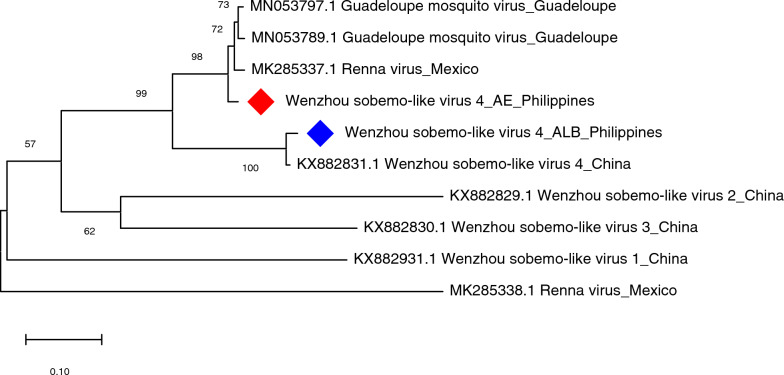
Molecular phylogenetic analysis of Wenzhou sobemo-like virus (WSLV4) from *Aedes aegypti* and *Aedes albopictus.* The evolutionary history was inferred by using the Maximum Likelihood method based on the Kimura 2-parameter model. Analysis of the hypothetical protein 2 involved four nucleotide sequences and a total of 414 positions in the final dataset. Values on the nodes represent bootstrap values based on 1000 bootstrap replicates; bootstrap values below 50% are not shown. The WSLV4 sequences from *Ae. aegypti* and *Ae. albopictus* in this study are labeled with a red and a blue rhombus, respectively

Wenzhou sobemo-like virus 4, *in silico*-detected in *Ae. aegypti* samples from Bagong Silang and in *Ae. albopictus* collected from Lalakay, was PCR-validated using primer sets WSLV4-H1–2 (for *Ae. aegypti* samples) and WSLV4-H2 (for *Ae. albopictus* samples). Capillary sequencing and BLAST results confirmed the identity of WSLV4 in *Ae. albopictus* samples [96–97% nucleotide (nt) identity with WSLV4 from China] but not in *Ae. aegypti* samples. WSLV4 identity in female adult *Ae. aegypti* samples was most similar to Renna virus (97% identity) after query in virus database, as presented in Fig. 13. Based on the WSLV4 tree constructed using the partial sequences of hypothetical protein 2, WSLV4-like contigs of *Ae. aegypti* and *Ae. albopictus* clustered separately. WSLV4-like contig of *Ae. albopictus* collected from Lalakay was most closely related to WSLV4 sequence from China with 100% maximum likelihood bootstrap values. However, WSLV4-like contig from *Ae. aegypti* clustered separately wherein it formed a clade (with 98% nodal support) with Renna virus from Mexico and Guadeloupe mosquito virus from Guadeloupe.

## Discussion

Virome analysis of *Ae. aegypti* and *Ae. albopictus* revealed 11 and 6 taxon groups, respectively. In concert with other studies, Phasi Charoen-like phasivirus (PCLV) and Humaita-Tubiacanga virus (HTV) dominated the metavirome of *Ae. aegypti* [34, 35]. Meanwhile, Wenzhou sobemo-like virus 4 (WSLV4) was the dominant virus in *Ae. albopictus* [36, 37].

Phasi Charoen-like phasivirus (PCLV), a bunyavirus from family *Phenuiviridae* and order *Bunyavirales* [38], was found to be widely distributed across all study sites from both *Ae. aegypti* and *Ae. albopictus*. This is the first study, as far as records show, that demonstrates the detection of near full-length genome (6603 bp, 3686 bp, 1360 bp for L, M, and S segments, respectively) of PCLV in *Ae. aegypti* collected in the Philippines. In addition, the presence of partial sequences of PCLV in *Ae. albopictus* from the Philippines is likewise first depicted here. First characterized from field-caught *Ae. aegypti* collected in Thailand, numerous studies from several other countries (Brazil, UK, China, USA, Australia, Guadeloupe, Ghana, and Grenada, among others) have shown detection of PCLV thereafter, which highly suggests its global distribution and prevalence in *Ae. aegypti* mosquitoes.

Humaita-Tubiacanga virus (HTV) is a positive-sense ssRNA virus named to reflect the origin of its host, *Ae. aegypti* from Humaita and Tubiacanga of Rio de Janeiro in Southeastern Brazil [39]. HTV is an unclassified virus but found to belong to a clade that branches basally to poleroviruses and sobemoviruses. In addition, previous studies identified HTV to be one of the three predominant insect-specific viruses identified in *Ae. aegypti* collected from Thailand and Northern Australia [35]. Upon sequence validation, only one primer set that targets the capsid gene of HTV from *Ae. aegypti* samples was able to produce successful amplification. No amplification was observed from all 30 samples where HTV RdRP was detected to be present. Since HTV-like contigs from *Ae. albopictus* only aligned to the RdRP gene of HTV, the presence of HTV in *Ae. albopictus* is still in question. Use of quantitative real-time PCR (qPCR) and design of another set of primers targeting HTV RdRP specific from *Ae. albopictus* could be done as the sensitivity of qPCR would allow validation of the presence of this virus even in low titers*.* However, *Ae. aegypti* is the only mosquito host to harbor HTV in the NCBI virus database out of all the submitted sequences from Brazil and Guadeloupe.

Wenzhou sobemo-like virus 4 (WSLV4), an unclassified RNA virus found in mosquitoes collected in China in 2013 [40], was detected in both *Aedes* species. Upon validation, WSLV4-like sequences from *Ae. albopictus* were confirmed to be WSLV4 (96–97% nt identity), whereas those from *Ae. aegypti* had 97% nucleotide identity to Renna virus (RENV; MK285337.1). Moreover, WSLV4-like sequences from *Ae. aegypti* also showed high nucleotide identity to another virus, Guadeloupe mosquito virus (GMV; MN053805.1). To further comprehend the discrepancy in the identity of the virus from *Ae. aegypti*, multiple sequence alignment of RENV, GMV, and WSLV4 was done, and results showed regions of WSLV4 being spanned by the other two viruses. Previous studies have shown this close relationship between WSLV4 and GMV [41], whereas RENV was shown to group phylogenetically with viruses identified from a variety of ticks and insects, including mosquitoes [42]. The phylogenetic analyses performed in the study confirmed this finding; analysis of the evolutionary relationship showed that WSLV4-like sequences from *Ae. aegypti* formed a distinct clade with RENV and GMV, whereas WSLV4-like sequences from *Ae. albopictus* clustered with WSLV4 from China. Further validation should be done, such as designing different primers that would differentiate RENV and GMV to assess the identity of WSLV4-like contig from *Ae. aegypti* and use of qPCR to detect the viruses if low amounts were present in the samples.

Anthropogenic habitat modifications have been shown to alter the biodiversity and ecological niche suitability of several mosquito species, resulting in changes to mosquito species diversity evenness. Such modifications may lead to the predominance of certain mosquito species, as some struggle to adapt, while generalist species, such as *Ae. aegypti* and *Ae. albopictus*, thrive [43]. As insect-specific viruses (ISVs), which dominated the virome profile of both *Aedes* spp. in this study, are not known to require maintenance and amplification by vertebrate hosts in nature, the prevalence of ISVs will then depend on the competence of mosquito spp. to harbor specific ISVs. Few viruses were identified in this study, with higher prevalence in *Ae. aegypti* collected from sites that have undergone land-use transformations. The observation that viruses were relatively higher in Lalakay and Bayog compared with Bagong Silang aligns with previous findings, suggesting that an increase in endemic viruses is more common in disturbed habitats, particularly human settlements, than in pristine primary forests. Furthermore, previous studies have demonstrated that mosquitoes found in disturbed habitats tend to carry more viral isolates compared with forest-dwelling mosquitoes [23, 24]. This phenomenon can be explained by the dilution effect hypothesis, which postulates that biodiversity loss may lead to a relative increase in disturbance-resilient, competent host species, thereby causing a relative increase in viral prevalence [44]. Furthermore, the higher virus prevalence in disturbed habitats could also be attributed to an effect known as the abundance effect. In this study, *Ae. aegypti* collection results were comparatively higher in disturbed habitats, aligning with previous findings that increased host abundance—rather than higher host infection rates—is a key determining factor in virus prevalence patterns [45]. Interestingly, even though 40% and 50% of the total viruses identified were present across all sample sites for *Ae. aegypti* and *Ae. albopictus,* respectively, no viruses were found to be exclusively shared between Bagong Silang and Lalakay, and Bagong Silang and Bayog alone. This observation could be attributed to the following: one, mosquito adults do not usually disperse far (< 500 m) from their larval niches [46]; and two, the lack or minimal transportation to and from Bagong Silang, the forest site, owing to its geographic location compared with the close association and availability of public transportation between Lalakay and Bayog.

As this study served to assess the impact that land use may have on virus distribution, a more in-depth study is warranted to better comprehend the links between land use, mosquito abundance, and viruses vectored by the mosquitoes for building predictive models of disease outbreaks. Other anthropogenic factors, such as food production changes (extreme livestock intensification without proper biosecurity measures) and trade and travel, should also be looked into, as analyses of recent emerging infectious diseases showed that these factors largely contribute to disease emergence [47, 48]. Lastly, a variety of other factors, which are largely independent of changes in land topography, should be considered, such as climatological conditions and high mutation frequency of viruses that could modify the virome of host species [49].

Results of this study also suggest predominantly vertical transmission of the important viruses. HTV and PCLV were detected from all sample types—reared adults and field-caught female and male adults. These two ISVs were already previously identified to be transmitted transovarially from previous studies [35, 39, 41, 42, 50, 51]. Meanwhile, CFAV was detected from reared adults, and just like other insect-specific flaviviruses, it circulates in natural mosquito populations, specifically, in mosquitoes of both sexes, suggesting vertical transmission. Moreover, it has been detected in or isolated from field-caught *Ae. aegypti* mosquitoes [15, 52, 53]. Merida virus (MERDV), on the other hand, was solely detected from field-collected male *Ae. aegypti.* This occurrence is supported by a previous report of MERDV being more compatible with vertical and venereal transmissions [54]. Most of the viruses identified in this study fall under the classification of vertically transmitted viruses (*n* = 8) since most of the samples processed were laboratory-emerged adult mosquitoes. However, it could be observed that field-collected male adults also share some of the viruses identified. Being nonhematophagous, they have most likely acquired the viruses through the vertical route. However, horizontal venereal transmission of viruses might also have played a role, as this type of transmission has been experimentally verified from previous studies, wherein males may acquire infection from females and vice versa [55–57]. These findings, in general, provide supporting evidence that vertical—and possibly horizontal venereal—transmission of viruses ensures the prevalence and survival of several viruses in their natural mosquito hosts [58, 59]. The likelihood that these viruses may have coexisted with their mosquito host for a long period of time and have evolved with them is also plausible [60].

Another important finding was the prevalence of ISVs and the nondetection of arboviruses in both *Aedes* species. Mosquito collection for this study was conducted in 2018, a year when fewer dengue cases were recorded nationwide compared with 2019. In Los Baños, Laguna, only 124 dengue cases were documented in 2018, whereas 588 cases were reported in 2019 [see Additional file 4]. This lower incidence of dengue during the time of collection may have resulted in the nondetection of DENV in the sampled mosquitoes from the target sites. Moreover, in this study, a conventional PCR and subsequent Sanger sequencing were done to validate only the next generation sequencing (NGS)-detected viruses. To verify the true presence or absence of known important viruses, RT-qPCR validation should be considered. Absence of some important arboviruses, especially DENV, could be further confirmed via RT-qPCR, as the technique allows more sensitive detection of viruses even at lower titers. Nevertheless, ISVs, which are capable of replicating in mosquito cells, but not in vertebrate cells, have been shown to be closely related to arboviruses that are of medical importance. This ISV-arbovirus relationship then instigates the question as to whether ISVs could play an important role in modulating arbovirus transmission.

Phasi Charoen-like phasivirus (PCLV), the predominant ISV in the metavirome of *Ae. aegypti*, has been previously shown to affect replication of some arboviruses. For instance, one study reported that dual infection with PCLV and cell-fusing agent virus (CFAV) in the *Ae. albopictus* cell line As23 inhibited the growth of ZIKV and DENV (both flaviviruses), as well as La Crosse virus (a bunyavirus) [61]. However, another study demonstrated no significant impact of persistent PCLV infection on replication and growth of a broader set of arboviral families: DENV and ZIKV (flaviviruses), Sindbis virus (alphavirus), and vesicular stomatitis virus (rhabdovirus) [62]. The contrasting results from these studies highlight the need to do *in vivo* studies or use live mosquitoes to further elucidate the potential use of PCLV as a biological control agent in altering the susceptibility of mosquitoes to certain pathogenic arboviruses. CFAV has also been the subject of interest owing to its mutually beneficial interactions with DENV in mosquito cells in culture [63]. One study has further shown that CFAV not only interacts with DENV in culture, but it also negatively interferes with both DENV type 1 and ZIKV *in vitro* and *in vivo* [15]. Overall, these studies contribute to the growing evidence that ISVs can interfere with arboviruses. Aside from the effect that these ISVs have on arboviruses, the possibility that ISVs could become a potential source of new arboviruses could not be ruled out, as already reviewed [18, 60, 64]. A closer inspection of the prevalence, ecology, and evolution of ISVs detected in this study is, therefore, warranted. Sampling mosquitoes from high-risk dengue areas during periods when cases are expected to rise should be considered. This approach would enable a better understanding of the tripartite dynamics among ISVs, arboviruses such as DENV, and their mosquito hosts in various epidemiological settings, ultimately providing valuable insights for designing effective control and prevention strategies for vector-borne viral diseases.

## Conclusions

Three study sites that differed in terms of land use revealed the diversity of viruses harbored by *Ae. aegypti* and *Ae. albopictus*. Viruses were relatively more diverse in Lalakay and Bayog, sites that have undergone land use transformations, compared with Bagong Silang, the forest site. Overall and for the first time, the work herein highlights the use of metagenomic sequencing in uncovering the virome composition of two of the most important vectors *Ae. aegypti* and *Ae. albopictus* collected in the Philippines*.* This study also provided insights as to how land use affects mosquito abundance and virus diversity. Lastly and most importantly, the prevalence of mosquito-specific viruses and the absence of arboviruses in the virome profile of both species instigate further studies on how such mosquito-specific viruses might influence the transmission of medically important arboviruses.

## Supplementary Information

Additional file 1. Supplementary Text S1. Immature mosquitoes were collected by using oviposition traps (ovitraps) and larval sampling. Supplementary Text S2. Results of molecular species identification of *Aedes aegypti *and *Aedes albopictus *samples. Supplementary Table S1. Capillary sequencing for species validation of *Aedes aegypti *and *Aedes albopictus *samples. Supplementary Fig. S1. Molecular phylogenetics analysis of *Aedes aegypti. * Supplementary Fig. S2. Molecular phylogenetics analysis of *Aedes albopictus.*

Additional file 2. Supplementary Table S2. *Aedes aegypti* and *Aedes albopictus* mosquito pools for virome sequencing. Supplementary Table S3. Anchored random primers and barcode DNA primers used in virome sequencing of *Aedes aegypti *and *Aedes albopictus. *Supplementary Table S4. Primers designed from viral contigs of *Aedes aegypti* and *Aedes albopictus*. Supplementary Table S5. Raw, cleaned, and filtered read counts per *Aedes aegypti *and *Aedes albopictus *sample set-up. Supplementary Table S6. Pooled assembly and annotation statistics from each *Aedes aegypti *and *Aedes albopictus *sample set-up.

Additional file 3. Supplementary Table S7. Summary of total assembled contigs per viral host identified from *Aedes aegypti *and *Aedes albopictus *samples. Supplementary Table S8. List of vertebrate-infecting viruses detected from *Aedes aegypti* and *Aedes albopictus*. Supplementary Table S9. Summary of viral families detected from *Aedes aegypti *and *Aedes albopictus *samples based on top hit contigs. Supplementary Table S10. Summary of PCR and capillary sequencing validation setups from *Aedes aegypti *and *Aedes albopictus *samples. Supplementary Table S11. Summary of capillary sequencing and BLAST results validating the presence of virus sequences detected from *Aedes aegypti* samples. Supplementary Table S12. Summary of capillary sequencing and BLAST results validating the presence of virus sequences detected from *Aedes albopictus* samples. Supplementary Table S13. Capillary sequencing and BLAST results validating the presence of Cell-fusing agent virus (CFAV) from *Aedes aegypti* samples. Supplementary Table S14. BLAST result of the longest CFAV-like contig detected from *Aedes aegypti*.

Additional file 4. Record of dengue cases in Laguna Province from 2010 to 2019

## Data Availability

The raw sequencing data were deposited under the BioProject No. PRJNA609581 and are available at the following URL:https://www.ncbi.nlm.nih.gov/sra/PRJNA609581). **Aedes aegypti** BioSample accession numbers are from SAMN48427844 to SAMN48427855; **Aedes albopictus** BioSample accession numbers are from SAMN48427856 to SAMN48427860.
