# Supplementary material for: Virome sequencing and analysis of Aedes aegypti and Aedes albopictus from ecologically different sites in the Philippines
Source: Parasit Vectors. 2025 Oct 24;18:426. doi: 10.1186/s13071-025-07073-7 (PMC12551354; doi:10.1186/s13071-025-07073-7)
Supplement: Supplementary file 1 — Additional file 1. Supplementary Text S1. Immature mosquitoes were collected by using oviposition traps (ovitraps) and larval sampling. Supplementary Text S2. Results of molecular species identification of Aedes aegypti and Aedes albopictus samples. Supplementary Table S1. Capillary sequencing for species validation of Aedes aegypti and Aedes albopictus samples. Supplementary Fig. S1. Molecular phylogenetics analysis of Aedes aegypti. Supplementary Fig. S2. Molecular phylogenetics analysis of Aedes albopictus. [file 13071_2025_7073_MOESM1_ESM.docx]

**Additional file 1: Text S1.** Immature mosquitoes were collected by using oviposition traps (ovitraps) and larval sampling.

Ovitrap

Ovitrapping was done during the month of August 2018. The total number of oviposition traps (ovitraps) that was set up is 200. For Bagong Silang, 50 ovitraps were distributed around households, while for Lalakay and Bayog, 75 ovitraps each were dispersed. After a week, ovitraps were collected from each household (10 ovitraps were unsuccessfully retrieved) and checked for presence of larvae. For two weeks of daily monitoring, adult mosquitoes that emerged, pupae, and fourth instar larvae were collected, identified using taxonomic keys, sorted according to species, and stored at -80°C.

Results of ovitrap collection showed high numbers of ovitrap positivity index (OPI). Highest OPI was observed for Lalakay (59.15%) with 42 positive ovitraps out of 71 that were collected from two districts. This was followed by Bayog, having an OPI of 49.30%, with 35 positive ovitraps out of 71 that were collected from four districts. And the lowest OPI was observed from Bagong Silang (37.50%), with 18 positive ovitraps out of 48 collected ovitraps.

Larval sampling

Larval sampling was done by surveying the three study sites for mosquito breeding habitats. It was done for one whole day at each site during the months of April, May, August, and October of 2018. Larvae were collected using plastic ladles, transferred to bowls for further sorting, and then using transfer pipettes, collected and stored into plastic tubes that contains the natural water source where they were obtained from. Afterwards, the collected larvae were brought to the laboratory, reared to fourth instar or adult for identification, and all were sorted according to species. Larvae were stored in TRIzol® reagent at -80 °C.

**Additional file 1: Text S2**. Results of molecular species identification of *Aedes aegypti* and *Aedes albpictus* samples.

Molecular analysis was done to confirm the species identity of both *Aedes* species, by targeting the mitochondrial cytochrome c oxidase subunit I (*COI*) and the nuclear ribosomal internal transcribed spacer 2 (*ITS2*) regions. Primers used for *COI* amplification were COI-MTFN (5’ GGATTTGGAAATTGATTAGTTCCTT 3’) and COI-MTRN (5’ AAAAATTTTAATTCCAGTTGGAACAGC 3’), while the primers used for *ITS2* were ITS2A (5’ TGTGAACTGCAGGACA 3’) and ITS2B (5’ TATGCTTAAATTCAGGGGGT 3’) [65,66].

Sanger sequencing and sequence alignments are shown in Table S1. Use of *COI* as gene marker resulted to 99.85-100% identity with *COI* of *Ae. aegypti* collected from India, Colombia, Cape Verde, and Japan. Meanwhile, *COI* of *Ae. albopictus* resulted to 99.1-99.79% identity with *COI* of the same species collected from Malaysia, Philippines, USA, and India. Using *ITS2* as gene marker, percent identities for *Ae. aegypti* were 98.73-100% and *ITS2* sequences were most similar with *Ae. aegypti* collected from USA, Russia, and Sri Lanka. Sequencing of *ITS2* of *Ae. albopictus* resulted to 98.72-98.95% nucleotide identity with *Ae. albopictus* collected from Palestine for two individuals while 93% identical with *Ae. albopictus* collected from Italy was observed for one individual. Overall, use of *COI* as gene marker supported the morphological identification done on *Ae. aegypti* and *Ae. albopictus.* Use of *ITS2* as second gene marker validated the species identity of *Ae. aegypti* as well, but for *Ae. albopictus,* only two out of the three individuals were confirmed (>98% nt identity). Compared to *COI,* a relatively higher level of variability of *ITS2* sequences among *Ae. albopictus* populations was observed as evident from a previous study.

Phylogenetic trees showing the relationship of *Ae. aegypti* and *Ae. albopictus* from the study with other similar species from Genbank based on *COI* **(Additional File 1: Fig. S1**) and *ITS2* (**Additional File 1: Fig. S2**) nucleotide sequences were constructed. Evolutionary relationships were inferred based on Tamura 3-parameter [67] and Kimura 2-parameter [68] models, respectively.

The results obtained from **Additional File 1: Fig. S1** indicate that, *Ae. aegypti* and *Ae. albopictus* samples from the study exhibited close evolutionary relationship with similar species from other countries based on *COI* sequences. Briefly, AE-1 and AE-3 formed a clade with *Ae. aegypti* from India, Cape Verde, Sri Lanka, and Japan with 99% nodal support whereas *Ae. aegypti* AE-2 was disclosed to the other two (AE-1 and -3) and more closely related to *Ae. aegypti* from Colombia (98% bootstrap value). Meanwhile, *Ae. albopictus* ALB-1 and ALB-2 shared a clade together with *Ae. albopictus* from the Philippines and Malaysia. *Ae. albopictus* ALB-3, on the contrary, formed a single group but still more closely related to the other two *Ae. albopictus* (ALB-1 and -2) from the study.

Phylogenetic trees constructed based on the *ITS2* gene corroborated the *COI* results as shown in **Additional File 1: Fig. S2**. *Ae. aegypti* AE-2 and AE-3 clustered in one group and were more closely related to *Ae. aegypti* located in Peru, Russia, and Sri Lanka. *Ae. aegypti* AE-1, on the other hand, clustered separately and more closely related to *Ae. aegypti* collected from Saudi Arabia, as well as *Ae. aegypti* located in USA. For *Ae. albopictus,* ALB-1 and -3 formed a group together with *Ae. albopictus* collected from Italy, China, and Palestine. *Ae. albopictus* ALB-2, even though clustered separately, is still more closely related to the other two *Ae. albopictus* (ALB-1 and -2) from the study.

Overall, these results further confirm the morphological identification of the collected *Aedes* mosquitoes. The close evolutionary relationship of *Ae. aegypti* and *Ae. albopictus* to similar species collected from various locations in several other countries provide evidence of the global spread of these vectors. However, a higher number of specimens of *Ae. aegypti* and *Ae. albopictus* analyzed using the *COI* gene would enable more conclusive analysis of the genetic diversity of the mosquitoes.

**Additional file 1: Table S1.** Summary of capillary sequencing results of *COI* and *ITS2* from *Aedes aegypti* and *Aedes albopictus* samples. AE- *Aedes aegypti* (three individuals, AE1-AE3); ALB- *Aedes albopictus* (three individuals, ALB1-ALB3)*.*

| Sample | Top Hits | Description | Accession | Query Cover | E-value | Identity | Country |  |
| --- | --- | --- | --- | --- | --- | --- | --- | --- |
| Gene marker: *COI* | | | | | | | | |
| AE_1 | 1 | *Aedes aegypti* | MK805535.1 | 100% | 0 | 100.00% | India |  |
|  | 2 | *Aedes aegypti* | MK805532.1 | 100% | 0 | 99.85% | India |  |
|  | 3 | *Aedes aegypti* | MK265726.1 | 100% | 0 | 99.85% | India |  |
| AE_2 | 1 | *Aedes aegypti* | KT766396.1 | 100% | 0 | 100.00% | Colombia |  |
|  | 2 | *Aedes aegypti* | KM203175.1 | 100% | 0 | 100.00% | Colombia |  |
|  | 3 | *Aedes aegypti* | KM203174.1 | 100% | 0 | 100.00% | Colombia |  |
| AE_3 | 1 | *Aedes aegypti* | MN019006.1 | 100% | 0 | 100.00% | Cape Verde |  |
|  | 2 | *Aedes aegypti* | LC482636.1 | 100% | 0 | 100.00% | Japan |  |
|  | 3 | *Aedes aegypti* | LC482630.1 | 100% | 0 | 100.00% | Japan |  |
| ALB_1 | 1 | *Aedes albopictus* | [KT211242.1](https://www.ncbi.nlm.nih.gov/nucleotide/KT211242.1?report=genbank&log$=nucltop&blast_rank=1&RID=3U87F1EY014) | 100% | 0 | 99.79% | Malaysia |  |
|  | 2 | *Aedes albopictus* | [KT211235.1](https://www.ncbi.nlm.nih.gov/nucleotide/KT211235.1?report=genbank&log$=nucltop&blast_rank=2&RID=3U87F1EY014) | 100% | 0 | 99.79% | Malaysia |  |
|  | 3 | *Aedes albopictus* | [KT211221.1](https://www.ncbi.nlm.nih.gov/nucleotide/KT211221.1?report=genbank&log$=nucltop&blast_rank=3&RID=3U87F1EY014) | 100% | 0 | 99.79% | Malaysia |  |
| ALB_2 | 1 | *Aedes albopictus* | [KY982339.1](https://www.ncbi.nlm.nih.gov/nucleotide/KY982339.1?report=genbank&log$=nucltop&blast_rank=1&RID=3U8KNCK1014) | 100% | 0 | 99.26% | Malaysia |  |
|  | 2 | *Aedes albopictus* | [KY982337.1](https://www.ncbi.nlm.nih.gov/nucleotide/KY982337.1?report=genbank&log$=nucltop&blast_rank=2&RID=3U8KNCK1014) | 100% | 0 | 99.26% | Malaysia |  |
|  | 3 | *Aedes albopictus* | [KX809764.1](https://www.ncbi.nlm.nih.gov/nucleotide/KX809764.1?report=genbank&log$=nucltop&blast_rank=3&RID=3U8KNCK1014) | 100% | 0 | 99.26% | Philippines |  |
| ALB_3 | 1 | *Aedes albopictus* | [MK372914.1](https://www.ncbi.nlm.nih.gov/nucleotide/MK372914.1?report=genbank&log$=nucltop&blast_rank=1&RID=3U8J4GEN016) | 100% | 0 | 99.25% | USA |  |
|  | 2 | *Aedes albopictus* | [DQ424959.1](https://www.ncbi.nlm.nih.gov/nucleotide/DQ424959.1?report=genbank&log$=nucltop&blast_rank=2&RID=3U8J4GEN016) | 99% | 0 | 99.25% | India |  |
|  | 3 | *Aedes albopictus* | [MF148291.1](https://www.ncbi.nlm.nih.gov/nucleotide/MF148291.1?report=genbank&log$=nucltop&blast_rank=3&RID=3U8J4GEN016) | 100% | 0 | 99.10% | Malaysia |  |
| Gene marker: *ITS2* | | | | | | | | |
| AE_1 | 1 | *Aedes aegypti* | KF471583.1 | 97% | 2.00E-112 | 98.73% | USA |  |
|  | 2 | *Aedes aegypti* | KF471582.1 | 97% | 2.00E-112 | 98.73% | USA |  |
|  | 3 | *Aedes aegypti* | KF471581.1 | 97% | 2.00E-112 | 98.73% | USA |  |
| AE_2 | 1 | *Aedes aegypti* | MH142327.1 | 99% | 8.00E-142 | 100.00% | Russia |  |
|  | 2 | *Aedes aegypti* | KY382418.1 | 99% | 8.00E-142 | 100.00% | Sri Lanka |  |
|  | 3 | *Aedes aegypti* | HE820724.1 | 99% | 8.00E-142 | 100.00% | Russia |  |
| AE_3 | 1 | *Aedes aegypti* | MH142327.1 | 99% | 4.00E-145 | 99.65% | Russia |  |
|  | 2 | *Aedes aegypti* | KY382418.1 | 99% | 4.00E-145 | 99.65% | Sri Lanka |  |
|  | 3 | *Aedes aegypti* | HE820724.1 | 99% | 4.00E-145 | 99.65% | Russia |  |
| ALB_1 | 1 | *Aedes albopictus* | MN062758.1 | 100% | 0 | 98.95% | Palestine |  |
|  | 2 | *Aedes albopictus* | MN062754.1 | 100% | 0 | 98.95% | Palestine |  |
|  | 3 | *Aedes albopictus* | MN062743.1 | 100% | 0 | 98.95% | Palestine |  |
| ALB_2 | 1 | *Aedes albopictus* | MN062758.1 | 100% | 0 | 98.72% | Palestine |  |
|  | 2 | *Aedes albopictus* | MN062754.1 | 100% | 0 | 98.72% | Palestine |  |
|  | 3 | *Aedes albopictus* | MN062743.1 | 100% | 0 | 98.72% | Palestine |  |
| ALB_3 | 1 | *Aedes albopictus* | KF471591.1 | 100% | 3.00E-172 | 93.38% | Italy |  |
|  | 2 | *Aedes albopictus* | KF471599.1 | 100% | 9.00E-172 | 93.36% | Italy |  |
|  | 3 | *Aedes albopictus* | KF471598.1 | 100% | 9.00E-172 | 93.36% | Italy |  |


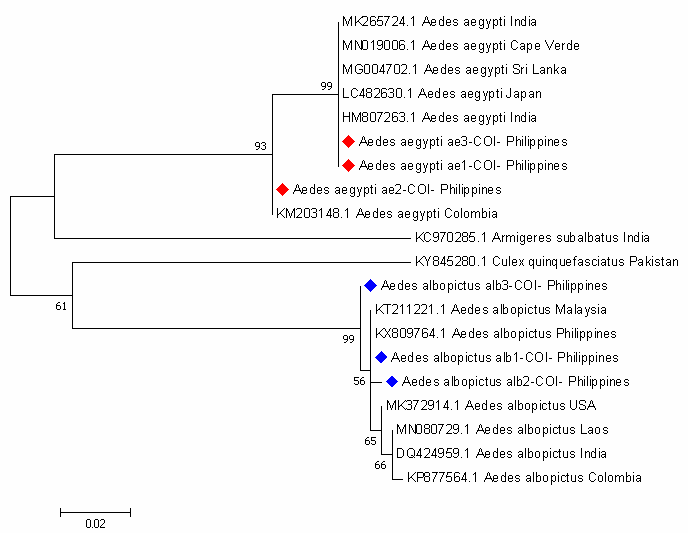


**Additional file 1: Fig. S1.** Molecular phylogenetic analysis of *Aedes aegypti* and *Aedes albopictus* based on *COI* nucleotide sequences. The evolutionary history was inferred by using the Maximum Likelihood method based on the Tamura 3-parameter model. The analysis involved 20 nucleotide sequences and a total of 343 positions in the final dataset. *Aedes aegypti* and *Aedes albopictus* from the study were labeled with red and blue rhombus, respectively. AE- *Aedes aegypti* (three individuals, AE1-AE3); ALB- *Aedes albopictus* (three individuals, ALB1-ALB3)*.*


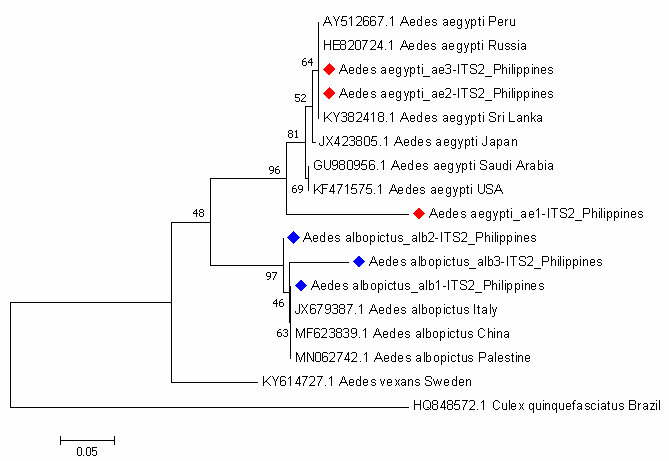


**Additional file 1: Fig. S2.** Molecular phylogenetic analysis of *Aedes aegypti* and *Aedes albopictus* based on *ITS2* nucleotide sequences. The evolutionary history was inferred by using the Maximum Likelihood method based on the Kimura 2-parameter model. The analysis involved 19 nucleotide sequences and a total of 179 positions in the final dataset. *Aedes aegypti* and *Aedes albopictus* from the study were labeled with red and blue rhombus, respectively. AE- *Aedes aegypti* (three individuals, AE1-AE3); ALB- *Aedes albopictus* (three individuals, ALB1-ALB3)*.*
