# Supplementary material for: Virome sequencing and analysis of Aedes aegypti and Aedes albopictus from ecologically different sites in the Philippines
Source: Parasit Vectors. 2025 Oct 24;18:426. doi: 10.1186/s13071-025-07073-7 (PMC12551354; doi:10.1186/s13071-025-07073-7)
Supplement: Supplementary file 2 — Additional file 2. Supplementary Table S2. Aedes aegypti and Aedes albopictus mosquito pools for virome sequencing. Supplementary Table S3. Anchored random primers and barcode DNA primers used in virome sequencing of Aedes aegypti and Aedes albopictus. Supplementary Table S4. Primers designed from viral contigs of Aedes aegypti and Aedes albopictus. Supplementary Table S5. Raw, cleaned, and filtered read counts per Aedes aegypti and Aedes albopictus sample set-up. Supplementary Table S6. Pooled assembly and annotation statistics from each Aedes aegypti and Aedes albopictus sample set-up. [file 13071_2025_7073_MOESM2_ESM.docx]

**Additional file 2: Table S2.** *Aedes aegypti* and *Aedes albopictus* mosquito pools for virome sequencing**.**

| Species | Site | Adult type | No. of individuals (pooled) | Sample name |
| --- | --- | --- | --- | --- |
| *Aedes aegypti* | Bagong Silang | Reared | 15 | AE-BS-R1 |
|  |  | Reared | 15 | AE-BS-R2 |
|  |  | Wild female | 15 | AE-BS-F |
|  |  | Wild male | 15 | AE-BS-M |
|  | Lalakay | Reared | 30 | AE-L-R1 |
|  |  | Reared | 50 | AE-L-R2 |
|  |  | Reared | 30 | AE-L-R3 |
|  |  | Reared | 30 | AE-L-R4 |
|  | Bayog | Reared | 15 | AE-B-R1 |
|  |  | Reared | 15 | AE-B-R2 |
|  |  | Wild female | 15 | AE-B-F |
|  |  | Wild male | 15 | AE-B-M |
| *Aedes albopictus* | Bagong Silang | Reared | 15 | ALB-BS-R1 |
|  |  | Reared | 15 | ALB-BS-R2 |
|  |  | Reared | 15 | ALB-BS-R3 |
|  | Lalakay | Reared | 15 | ALB-L-R1 |
|  |  | Reared | 15 | ALB-L-R2 |

BS- Bagong Silang, L- Lalakay, B- Bayog (collection sites); F- female, M- male (sex); Reared- mosquito eggs/larvae collected using ovitrap/larval sampling that were reared to adult in the laboratory, Wild- field-caught adult mosquitoes sampled in selected sites using sweep net (adult type).

**Additional file 2: Table S3.** Anchored random primers and barcode DNA primers used in virome sequencing of *Aedes aegypti* and *Aedes albopictus.* Barcode primers were anchored to random hexamers to facilitate reverse transcription and the succeeding second strand synthesis.

| Primer type | Primer number | Primers (5′-3′) |
| --- | --- | --- |
| Anchored random primers (RT) | RT1 | GCCGGAGCTCTGCAGATATCNNNNNN |
|  | RT2 | GTATCGCTGGACACTGGACCNNNNNN |
|  | RT3 | ATCGTCGTCGTAGGCTGCTCNNNNNN |
|  | RT4 | CGTAGATAAGCGGTCGGCTCNNNNNN |
|  | RT5 | CATCACATAGGCGTCCGCTGNNNNNN |
|  | RT6 | CGCAGGACCTCTGATACAGGNNNNNN |
|  | RT7 | CGTCCAGGCACAATCCAGTCNNNNNN |
|  | RT8 | CCGAGGTTCAAGCGAGGTTGNNNNNN |
|  | RT9 | ACGGTGTGTTACCGACGTCCNNNNNN |
|  | RT10 | CGACCCTCTTATCGTGACGGNNNNNN |
|  | RT11 | GAGCCCCTAGACACAACGACNNNNNN |
|  | RT12 | GGTGGGCGTGTGAAATCGACNNNNNN |
|  | RT13 | GAAAATGAGAGGGGAGGCGGNNNNNN |
| Barcode primers (BP) | BP1 | GCCGGAGCTCTGCAGATATC |
|  | BP2 | GTATCGCTGGACACTGGACC |
|  | BP3 | ATCGTCGTCGTAGGCTGCTC |
|  | BP4 | CGTAGATAAGCGGTCGGCTC |
|  | BP5 | CATCACATAGGCGTCCGCTG |
|  | BP6 | CGCAGGACCTCTGATACAGG |
|  | BP7 | CGTCCAGGCACAATCCAGTC |
|  | BP8 | CCGAGGTTCAAGCGAGGTTG |
|  | BP9 | ACGGTGTGTTACCGACGTCC |
|  | BP10 | CGACCCTCTTATCGTGACGG |
|  | BP11 | GAGCCCCTAGACACAACGAC |
|  | BP12 | GGTGGGCGTGTGAAATCGAC |
|  | BP13 | GAAAATGAGAGGGGAGGCGG |

**Additional file 2: Table S4.** Primers designed from viral contigs of *Aedes aegypti* and *Aedes albopictus*.

| Virus  (Family) | Primer Name | Target gene | Primers (5′-3′) | Target size (bp) |
| --- | --- | --- | --- | --- |
| Cell fusing agent virus, CFAV  (*Flaviviridae)* | CFAV-P1 | polyprotein | F: GCACAACCAAGCTAACACCC | 482 |
|  |  |  | R: CCCGGTCCATGAGCTTTGAA |  |
|  | CFAV-P2 | polyprotein | F: GATTCCCTTCAGGGCTGTCC | 408 |
|  |  |  | R: AGCAACCCTATGGCAGACAC |  |
| Humaita-Tubiacanga virus, HTV  (unassigned) | HTV-R | RNA-dependent RNA polymerase | F: AGAACTCACACACTGAGCCG | 591 |
|  |  |  | R: AGCGGATTTTTCCGCGAGTA |  |
|  | HTV-C | capsid | F: CCGGATGTGCGACCACTTAT | 435 |
|  |  |  | R: CACCACCGGAGTGCATCTTA |  |
| Merida virus, MERDV  (*Rhabdoviridae)* | MERDV-R | RNA-dependent RNA polymerase | F: GGGAGAGTTCAACCTGCGAATA | 585 |
|  |  |  | R: ATCTTCCCGTGAGGTCGGAG |  |
|  | MERDV-Np | nucleoprotein | F: GCCAGTGTGAACCCTTACCT | 491 |
|  |  |  | R: TAACCTTCTGGGTCTCTGAGG |  |
| Phasi Charoen-like phasivirus, PCLV  (*Phenuiviridae*) | PCLV-R | RNA-dependent RNA polymerase | F: AAACGCATCCGCACTTCATT | 534 |
|  |  |  | R: TCTGAGGAGAAGTTAGGCCC |  |
|  | PCLV-G | glycoprotein precursor | F: CTGTGTCAAGCAAGGACGGG | 551 |
|  |  |  | R: ACCACAATTAGCACAGCCTCT |  |
|  | PCLV-Nc | nucleocapsid | F: ATAAATCGACAAGATTGACTCCAC | 570 |
|  |  |  | R: GTTTGCATCTGACGTGGGAA |  |
| Wenzhou sobemo-like virus 4, WSLV4  (unclassified) | WSLV4-H1-2 | hypothetical protein 1 and 2 | F: GGACCTATTCCGCATGGAGG | 439 |
|  |  |  | R: CTCCACCCGATGAGACATGG |  |
|  | WSLV4-H1 | hypothetical protein 1 | F: AGCTGTTAATCGTAATGGCAGTG | 429 |
|  |  |  | R: TCGCTTTAGACAAGCCCACC |  |
| Hubei mosquito virus 2 (HMV2)  (unclassified) | HMV2-H1a | hypothetical protein 1 | F: TGGGTCCAACGCTTCCCTAC | 415 |
|  |  |  | R: CGAATGGCTTGGCTGTATAAACTA |  |
|  | HMV2-H1b | hypothetical protein 1 | F: CGAATGGCTTGGCTGTATAAACTA | 415 |
|  |  |  | R: TGGGTCCAACGCTTCCCTAC |  |

Additional file 2: Table S5. Raw, cleaned, and filtered read counts per *Aedes aegypti* and *Aedes albopictus* sample set-up.

| Sample | Raw reads | Cleaned reads | % cleaned | Filtered reads | % filtered |
| --- | --- | --- | --- | --- | --- |
| AE-BS-R1_a | 78,459 | 45,245 | 57.67 | 40,925 | 90.45 |
| AE-BS-R1_b | 66,570 | 38,395 | 57.68 | 26,007 | 67.74 |
| AE-BS-R2_a | 212,858 | 133,894 | 62.90 | 89,126 | 66.56 |
| AE-BS-R2_b | 168,231 | 86,402 | 51.36 | 63,066 | 72.99 |
| AE-BS-F1 | 182,664 | 105,243 | 57.62 | 96,986 | 92.15 |
| AE-BS-F2 | 369,855 | 308,930 | 83.53 | 307,404 | 99.51 |
| AE-BS-M1 | 83,351 | 46,241 | 55.48 | 43,826 | 94.78 |
| AE-BS-M2 | 57,128 | 28,884 | 50.56 | 25,098 | 86.89 |
| AE-L-R1_a | 226,112 | 214,836 | 95.01 | 101,984 | 47.47 |
| AE-L-R1_b | 177,744 | 159,908 | 89.97 | 136,183 | 85.16 |
| AE-L-R1_c | 441,869 | 427,475 | 96.74 | 257,023 | 60.13 |
| AE-L-R1_d | 388,506 | 381,600 | 98.22 | 158,848 | 41.63 |
| AE-L-R2_a | 289,024 | 262,026 | 90.66 | 179,778 | 68.61 |
| AE-L-R2_b | 100,959 | 87,108 | 86.28 | 76,777 | 88.14 |
| AE-L-R2_c | 161,665 | 138,755 | 85.83 | 127,993 | 92.24 |
| AE-L-R2_d | 234,787 | 208,645 | 88.87 | 167,123 | 80.10 |
| AE-B-R1_a | 179,330 | 92,926 | 51.82 | 92,331 | 99.36 |
| AE-B-R1_b | 273,836 | 163,434 | 59.68 | 148,193 | 90.67 |
| AE-B-R2_a | 204,799 | 102,497 | 50.05 | 73,869 | 72.07 |
| AE-B-R2_b | 253,309 | 134,768 | 53.20 | 126,533 | 93.89 |
| AE-B-F1 | 155,393 | 135,202 | 87.01 | 89,447 | 66.16 |
| AE-B-F2 | 164,307 | 72,454 | 44.10 | 69,716 | 96.22 |
| AE-B-M1 | 232,410 | 117,832 | 50.70 | 55,281 | 46.92 |
| AE-B-M2 | 429,469 | 322,592 | 75.11 | 230,816 | 71.55 |
| Total | 5,132,635 | 3,815,292 | 74.33 | 2,833,071 | 74.26 |
| ALB-BS-R1_a | 138,663 | 77,646 | 56.00 | 72,883 | 93.87 |
| ALB-BS-R1_b | 198,832 | 107,114 | 53.87 | 95,498 | 89.16 |
| ALB-BS-R1_c | 169,890 | 92,690 | 54.56 | 84,587 | 91.26 |
| ALB-BS-R2_a | 121,224 | 67,191 | 55.43 | 47,547 | 70.76 |
| ALB-BS-R2_b | 146,700 | 77,902 | 53.10 | 62,108 | 79.73 |
| ALB-BS-R2_c | 187,505 | 104,966 | 55.98 | 74,931 | 71.39 |
| ALB-L-R1_a | 254,202 | 180,679 | 71.08 | 41,121 | 22.76 |
| ALB-L-R1_b | 221,255 | 170,061 | 76.86 | 39,796 | 23.40 |
| ALB-L-R2_a | 178,548 | 96,982 | 54.32 | 74,197 | 76.51 |
| ALB-L-R2_b | 181,369 | 93,070 | 51.32 | 78,089 | 83.90 |
| Total | 1,798,188 | 1,068,301 | 59.41 | 670,757 | 62.79 |

Raw, cleaned (≥ Q15), and filtered (removal of mosquito host) clean reads are tallied per *Aedes aegypti* and *Aedes albopictus* sample set-up. Percentage of reads retained are also detailed after cleaning and filtering of reads. Mosquito sample: AE- *Aedes aegypt****i***; ALB- *Aedes albopictus*. Collection sites: BS- Bagong Silang; L-Lalakay; B- Bayog. Adult type: R- mosquito eggs/larvae collected using ovitrap/larval sampling that were reared to adult in the laboratory; F – wild/field-caught female adults sampled in selected sites using sweep net; M – wild/field-caught male adults sampled in selected sites using sweep net. Viral purification: 1- semi-pure samples; 2 – ultra-pure samples. Letters indicate replicate set-ups.

**Additional file 2: Table S6**. Pooled assembly and annotation statistics from each *Aedes aegypti* and *Aedes albopictus* sample set-up.

| Sample | Contigs no. | Contigs no. ≥ 100 bp | Largest contig* | N50* | Viral contigs |
| --- | --- | --- | --- | --- | --- |
| AE-BS-R1 | 3460 | 475 | 2374 | 135 | 160 |
| AE-BS-R2 | 3907 | 695 | 2182 | 159 | 655 |
| AE-BS-F1 | 3946 | 1529 | 2752 | 157 | 669 |
| AE-BS-F2 | 2026 | 988 | 3301 | 422 | 2214 |
| AE-BS-M1 | 1941 | 260 | 2684 | 161 | 97 |
| AE-BS-M2 | 2049 | 214 | 3145 | 192 | 140 |
| AE-L-R1 | 37584 | 6496 | 1877 | 131 | 15935 |
| AE-L-R2 | 31193 | 6121 | 1044 | 134 | 4792 |
| AE-B-R1 | 581 | 239 | 4097 | 1179 | 136 |
| AE-B-R2 | 2087 | 426 | 2694 | 189 | 482 |
| AE-B-F1 | 2601 | 296 | 2096 | 183 | 372 |
| AE-B-F2 | 3376 | 373 | 2016 | 159 | 106 |
| AE-B-M1 | 1271 | 398 | 2902 | 218 | 166 |
| AE-B-M2 | 6206 | 608 | 3192 | 288 | 1698 |
| Average | 7,302.00 | 1,365.57 | 2,596.86 | 264.79 | - |
| Total | 102,228 | 19,118 | - | - | 27622 |
| ALB-BS-R1 | 9052 | 1058 | 1219 | 133 | 288 |
| ALB-BS-R2 | 13649 | 1925 | 982 | 107 | 412 |
| ALB-L-R1 | 1635 | 287 | 2250 | 188 | 187 |
| ALB-L-R2 | 4798 | 1202 | 2603 | 150 | 77 |
| Average | 7,283.50 | 1,118.00 | 1,763.50 | 144.50 | - |
| Total | 29,134 | 4,472 | - | - | 964 |

Collection sites: BS- Bagong Silang; L-Lalakay; B- Bayog. Adult type: R- mosquito eggs/larvae collected using ovitrap/larval sampling that were reared to adult in the laboratory; F – wild/field-caught female adults sampled in selected sites using sweep net; M – wild/field-caught male adults sampled in selected sites using sweep net. Viral purification: 1- semi-pure samples; 2 – ultra-pure samples. *Based on contigs ≥ 100 bp.
