# Supplementary material for: Virome sequencing and analysis of Aedes aegypti and Aedes albopictus from ecologically different sites in the Philippines
Source: Parasit Vectors. 2025 Oct 24;18:426. doi: 10.1186/s13071-025-07073-7 (PMC12551354; doi:10.1186/s13071-025-07073-7)
Supplement: Supplementary file 3 — Additional file 3. Supplementary Table S7. Summary of total assembled contigs per viral host identified from Aedes aegypti and Aedes albopictus samples. Supplementary Table S8. List of vertebrate-infecting viruses detected from Aedes aegypti and Aedes albopictus. Supplementary Table S9. Summary of viral families detected from Aedes aegypti and Aedes albopictus samples based on top hit contigs. Supplementary Table S10. Summary of PCR and capillary sequencing validation setups from Aedes aegypti and Aedes albopictus samples. Supplementary Table S11. Summary of capillary sequencing and BLAST results validating the presence of virus sequences detected from Aedes aegypti samples. Supplementary Table S12. Summary of capillary sequencing and BLAST results validating the presence of virus sequences detected from Aedes albopictus samples. Supplementary Table S13. Capillary sequencing and BLAST results validating the presence of Cell-fusing agent virus (CFAV) from Aedes aegypti samples. Supplementary Table S14. BLAST result of the longest CFAV-like contig detected from Aedes aegypti. [file 13071_2025_7073_MOESM3_ESM.docx]

**Additional file 3: Table S7.** Summary of total assembled contigs per viral host identified from *Aedes aegypti* and *Aedes albopictus* samples.

| Mosquito species | Viral Host | No. of assembled contigs |
| --- | --- | --- |
| *Aedes aegypti* | vertebrate virus | 556 |
|  | insect virus | 27061 |
|  | plant virus | 2 |
|  | unidentified | 74606 |
| *Aedes albopictus* | vertebrate virus | 381 |
|  | insect virus | 567 |
|  | plant virus | 4 |
|  | environmental sample | 12 |
|  | unidentified | 28170 |

**Additional file 3: Table S8.** List of vertebrate-infecting viruses detected from *Aedes aegypti* and *Aedes albopictus*.

| *Aedes aegypti* | *Aedes albopictus* |
| --- | --- |
| Bluetongue virus  Chicken picornavirus UCC/PhV  Cynomolgus cytomegalovirus (unverified)  Felid alphaherpesvirus 1  Human gammaherpesvirus 4  Human pegivirus 2  Human picobirnavirus  Influenza A virus (A/Uruguaiana/LACENRS-296/2016(H1N1))  Lassa mammarenavirus  Norovirus GII  Rotavirus C (unverified)  Stealth virus 1 | Cynomolgus cytomegalovirus (unverified)  Human gammaherpesvirus 4  Norovirus GII  Stealth virus 1 |

**Additional file 3: Table S9.** Summary of viral families detected from from *Aedes aegypti* and *Aedes albopictus* samples based on top hit contigs.

| *Aedes aegypti* | Count | *Aedes albopictus* | Count |
| --- | --- | --- | --- |
| *Arenaviridae* | 1 | *Calciviridae* | 1 |
| *Calciviridae* | 1 | *Endornaviridae* | 1 |
| *Flaviviridae* | 3 | *Flaviviridae* | 1 |
| *Herpesviridae* | 4 | *Herpesviridae* | 3 |
| *Orthomyxoviridae* | 1 | *Phenuiviridae* | 1 |
| *Peribunyaviridae* | 1 | *unclassified* | 4 |
| *Phenuiviridae* | 1 | Total | 11 |
| *Picornaviridae* | 1 |  |  |
| *Reoviridae* | 2 |  |  |
| *Rhabdoviridae* | 1 |  |  |
| *unclassified* | 3 |  |  |
| Total | 19 |  |  |

**Additional file 3: Table S10.** Summary of PCR and capillary sequencing validation setups from *Aedes aegypti* and *Aedes albopictus* samples.

| Virus | Primer Name | Target region | PCR # / PCR + / CapSeq # / CapSeq + | | | | |
| --- | --- | --- | --- | --- | --- | --- | --- |
|  |  |  | *Aedes aegypti* | | | *Aedes albopictus* | |
|  |  |  | BS  (N=8) | L  (N=8) | B  (N=8) | BS  (N=6) | L  (N=4) |
| Cell fusing agent virus | CFAV-P1 | polyprotein | - | 8/5/4/3 | 1/1/1/- | - | - |
|  | CFAV-P2 | polyprotein | - | 8/7/5/4 | 1/1/1/1 | - | - |
| Humaita-Tubiacanga virus | HTV-R | RdRP | 8/0/-/- | 8/0/-/- | 8/0/-/- | 6/0/-/- | 4/0/-/- |
|  | HTV-C | capsid | 8/8/8/8 | 8/8/8/8 | 8/8/8/8 | 6/0/-/- | 4/0/-/- |
| Merida virus | MERDV-R | RdRP | - | - | 2/2/2/2 | - | - |
|  | MERDV-Np | nucleoprotein | - | - | 2/0/-/- | - | - |
| Phasi Charoen-like phasivirus | PCLV-R | RdRP | 8/8/8/8 | 8/8/6/6 | 8/8/7/7 | 3/2/1/1 | 4/3/3/3 |
|  | PCLV-G | glycoprotein | 8/8/8/8 | 8/8/8/8 | 8/8/8/8 | 6/2/1/1 | 4/0/-/- |
|  | PCLV-Nc | nucleocapsid | 8/8/8/8 | 8/7/7/7 | 8/7/7/7 | 3/0/-/- | - |
| Stealth virus 1 | SV1-Gs1 | genomic sequence | 6/0/-/- | 4/0/-/- | 2/1/1/1 | 3/0/-/- | 2/0/-/- |
|  | SV1-Gs2 | genomic sequence | 6/2/1/1 | 8/7/4/4 | 2/1/1/1 | 3/2/2/2 | 2/1/1/1 |
| Wenzhou sobemo-like virus 4 | WSLV4-H1-2 | hypothetical protein 1 and 2 | 2/2/2/0 | - | - | - | 4/0/-/- |
|  | WSLV4-H1 | hypothetical protein 1 | 2/0/-/- | - | - | - | 4/3/2/2 |
| Hubei mosquito virus 2 | HMV2-H1a | hypothetical protein 1 | - | - | - | - | 4/4/0/- |
|  | HMV2-H1b | hypothetical protein 1 | - | - | - | - | 4/4/4/4 |
| TOTAL | | | 174/ 123/ 113/ 108 | | | 66/ 21/ 14/ 14 | |

Number of PCR set-ups based on bioinformatics results/Number of PCR positive (detectable) amplicons/Number of samples submitted for capillary sequencing/Number of samples with confirmed identity based on capillary sequencing results. BS=Bagong Silang, L=Lalakay, B=Bayog (collection sites). N=total number of samples per set-up.

**Additional file 3: Table S11**. Summary of capillary sequencing and BLAST results validating the presence of virus sequences detected from *Aedes aegypti* samples. Nucleotide sequences were queried against reference viral database (RVDBv16.0). F- Forward; R-Reverse.

| Virus | Primer  (target region) |  | BLAST Top Hit | Query cover (%) | E-value | Identity (%) | Accession No. | Location |
| --- | --- | --- | --- | --- | --- | --- | --- | --- |
| Cell fusing agent virus  # of sample: 8 | CFAV-P1  (polyprotein) | F | Cell fusing agent virus isolate CC_A_CFAV polyprotein gene, partial cds;  Flavivirus cell fusing agent polyprotein gene, | 99 | 0 | 97 | MH310082.1;  M91671.1 | USA |
|  |  | R | Cell fusing agent virus isolate CC_A_CFAV polyprotein gene, partial cds | 100 | 0 | 97 | MH310082.1 | USA |
|  | CFAV-P2  (polyprotein) | F | Cell fusing agent virus strain Galveston | 98-100 | 8E-153  to  2E-142 | 95-99 | NC_001564.2 | USA |
|  |  | R | Cell fusing agent virus strain Galveston;  Cell fusing agent virus strain Rio Piedras02 polyprotein gene, partial cds | 97-100 | 6E-155  to  6E-148 | 96-98 | NC_001564.2;  GQ165810.1 | USA;  Puerto Rico |
| Humaita-Tubiacanga virus  # of sample: 24 | HTV-C  (capsid) | F | Humaita-Tubiacanga virus isolate Rio, capsid gene | 96-100 | 0 to  9E-154 | 94-99 | KR003802.1 | Brazil |
|  |  | R | Humaita-Tubiacanga virus isolate Rio, capsid gene | 95-99 | 0 to  4E-163 | 97-99 | KR003802.1 | Brazil |
| Merida virus  # of sample: 2 | MERDV-Np  (nucleoprotein) | F | Merida virus isolate MERD-Mex07 | 98-99 | 0 | 94-95 | NC_040599.1 | Mexico |
|  |  | R | Merida virus isolate CC_H;  Merida virus isolate MERD-Mex07 | 99-100 | 0 | 95-96 | MH310083.1;  NC_040599.1 | USA;  Mexico |
| Phasi Charoen-like phasivirus  # of sample: 21 | PCLV-R  (L/RdRP) | F | Phasi Charoen-like phasivirus strain Zhanjiang01 segment L;  Phasi Charoen-like phasivirus RNA-dependent RNA polymerase gene;  Phasi Charoen-like phasivirus isolate CC_A_PCLV segment L. | 41-100 | 0 to  1E-30 | 75-97 | MF614132.1;  KM001085.1;  MH310079.1 | China;  Thailand;  USA |
|  |  | R | Phasi Charoen-like phasivirus strain Zhanjiang01 segment L;  Phasi Charoen-like phasivirus RNA-dependent RNA polymerase gene;  Phasi Charoen-like phasivirus isolate Rio segment L RNA-dependent RNA polymerase gene, | 38-100 | 0 to 9E-33 | 76-97 | MF614132.1;  KM001085.1;  NC_038262.1 | China;  Thailand;  Brazil |
| Phasi Charoen-like phasivirus  # of sample: 24 | PCLV-G  (M/glycoprotein precursor) | F | Phasi Charoen-like phasivirus strain 2b segment M | 97-100 | 0 to 5E-169 | 94-98 | MH237598.1 | Australia |
|  |  | R | Phasi Charoen-like phasivirus strain 2b segment M;  Phasi Charoen-like phasivirus glycoprotein precursor, gene, | 94-100 | 0 | 96-98 | MH237598.1;  KM001086.1 | Australia; Thailand |
| Phasi Charoen-like phasivirus  # of sample: 22 | PCLV-Nc  (S/nucleocapsid) | F | Phasi Charoen-like phasivirus nucleocapsid gene;  Phasi Charoen-like phasivirus isolate Rio segment S, nucleocapsid gene; | 92-100 | 0 to 6E-129 | 95-97 | KM001087.1;  NC_038263.1; | Thailand;  Brazil; |
|  |  | R | Phasi Charoen-like phasivirus nucleocapsid gene;  Phasi Charoen-like phasivirus isolate Rio segment S, nucleocapsid gene; | 95-100 | 0 to 2E-54 | 93-98 | KM001087.1;  NC_038263.1; | Thailand;  Brazil; |
| Wenzhou sobemo-like virus 4  # of sample: 2 | WSLV4-H1-2  (hypothetical protein 1 and 2) | F | Renna virus clone RENV_S1_Mex_2016 | 100 | 1.00E-177 to 1.00E-176 | 97 | MK285337.1 | Mexico |
|  |  | R | Renna virus clone RENV_S1_Mex_2016 | 100 | 0 | 97 | MK285337.1 | Mexico |

**Additional file 3: Table S12.** Summary of capillary sequencing and BLAST results validating the presence of virus sequences detected from *Aedes albopictus* samples. Nucleotide sequences were queried against reference viral database (RVDBv16.0). F- Forward; R-Reverse.

| Virus to | Primer  (target region) |  | BLAST Top Hit | Query cover (%) | E-value | Identity (%) | Accession No. | Location |
| --- | --- | --- | --- | --- | --- | --- | --- | --- |
| Phasi Charoen-like phasivirus  # of sample: 4 | PCLV-R  (L/RdRP) | F | Phasi Charoen-like phasivirus strain Zhanjiang01 segment L;  Phasi Charoen-like phasivirus RNA-dependent RNA polymerase gene;  Phasi Charoen-like phasivirus isolate CC_A_PCLV segment L | 47-100 | 0 to 1.00E-43 | 83-97 | MF614132.1;  KM001085.1;  MH310079.1 | China;  Thailand;  USA |
|  |  | R | Phasi Charoen-like phasivirus strain Zhanjiang01 segment L;  Phasi Charoen-like phasivirus RNA-dependent RNA polymerase gene;  Phasi Charoen-like phasivirus isolate Rio segment L RNA-dependent RNA polymerase gene, | 53-100 | 0 to 2.00E-59 | 85-97 | MF614132.1;  KM001085.1;  NC_038262.1 | China;  Thailand;  Brazil |
| Phasi Charoen-like phasivirus (glycoprotein precursor gene)  # of sample: 1 | PCLV-G  (M/glycoprotein precursor) | F | Phasi Charoen-like phasivirus glycoprotein precursor | 100 | 0 | 97 | KM001086.1 | Thailand |
|  |  | R | Phasi Charoen-like phasivirus glycoprotein precursor | 100 | 0 | 97 | KM001086.1 | Thailand |
| Hubei mosquito virus 2  # of sample: 4 | HMV2-H1b  (hypothetical protein 1) | F | Hubei mosquito virus 2 strain spider133708 segment 2 hypothetical protein 1 gene | 99-100 | 2E-47 to 7E-40 | 73-74 | KX882874.1 | China |
|  |  | R | Hubei mosquito virus 2 strain spider133708 segment 2 hypothetical protein 1 gene | 99-100 | 2E-47 to 7E-40 | 73-74 | KX882874.1 | China |
| Wenzhou sobemo-like virus 4  # of sample: 2 | WSLV4-H2  (hypothetical protein 2) |  | Wenzhou sobemo-like virus 4 strain mosZJ35391 hypothetical protein 1 and hypothetical protein 2 genes | 99-100 | 2.00E-180 to 1.00E-177 | 97 | NC_033138.1 | China |
|  |  |  | Wenzhou sobemo-like virus 4 strain mosZJ35391 hypothetical protein 1 and hypothetical protein 2 genes | 100 | 3.00E-179 to 5.00E-176 | 96 | NC_033138.1 | China |

**Additional file 3: Table S13.** Capillary sequencing and BLAST results validating the presence of *Cell fusing agent* *virus* (CFAV) from *Aedes aegypti* samples. Nucleotide sequences were queried against reference viral database (RVDBv16.0).

| Sample code | Primer | Blast Top Hit | Query cover (%) | E-value | Identity (%) | Accession No. |
| --- | --- | --- | --- | --- | --- | --- |
| AE-L-R_a_rep1 | CFAV-b-F | Cell fusing agent virus strain Galveston, complete genome | 99 | 3e-165 | 97 | NC_001564.2 |
|  | CFAV-b-R | Cell fusing agent virus strain Galveston, complete genome | 99 | 4e-163 | 97 | NC_001564.2 |
| AE-L-R_a_rep2 | CFAV-b-F | Cell fusing agent virus strain Galveston, complete genome | 99 | 3e-159 | 97 | NC_001564.2 |
|  | CFAV-b-R | Cell fusing agent virus strain Galveston, complete genome | 99 | 2e-161 | 97 | NC_001564.2 |
| AE-L-R_b_rep1 | CFAV-b-F | Cell fusing agent virus strain Galveston, complete genome | 99 | 2e-173 | 98 | NC_001564.2 |
|  | CFAV-b-R | Cell fusing agent virus strain Galveston, complete genome | 99 | 2e-168 | 98 | NC_001564.2 |
| AE-L-R_b_rep2 | CFAV-a-F | Cell fusing agent virus isolate CC_A_CFAV polyprotein gene, partial cds. | 100 | 0 | 95 | MH310082.1 |
|  | CFAV-a-R | Cell fusing agent virus isolate CC_A_CFAV polyprotein gene, partial cds. | 100 | 8e-91 | 80 | MH310082.1 |
| AE-L-R_c_rep1 | CFAV-a-F | Flavivirus cell fusing agent polyprotein gene, complete cds. | 100 | 0 | 95 | M91671.1 |
|  | CFAV-a-R | Cell fusing agent virus isolate CC_A_CFAV polyprotein gene, partial cds. | 100 | 2e-156 | 88 | MH310082.1 |
| AE-L-R_c_rep2 | CFAV-a-F | Flavivirus cell fusing agent polyprotein gene, complete cds. | 100 | 0 | 95 | M91671.1 |
|  | CFAV-a-R | Cell fusing agent virus isolate CC_A_CFAV polyprotein gene, partial cds. | 100 | 0 | 97 | MH310082.1 |
| AE-L-R_d_rep2 | CFAV-b-F | Cell fusing agent virus strain Galveston, complete genome | 100 | 2e-173 | 97 | NC_001564.2 |
|  | CFAV-b-R | Cell fusing agent virus strain Rio Piedras02 polyprotein gene, partial cds. | 99 | 1e-157 | 97 | GQ165810.1 |
| AE-B-F_rep2 | CFAV-b-F | Cell fusing agent virus strain Galveston, complete genome | 99 | 9e-166 | 97 | NC_001564.2 |
|  | CFAV-b-R | Cell fusing agent virus strain Rio Piedras02 polyprotein gene, partial cds. | 100 | 6e-161 | 97 | GQ165810.1 |

Mosquito sample: AE- *Aedes aegypti*. Collection sites: BS- Bagong Silang; L-Lalakay; B- Bayog. Adult type: R- mosquito eggs/larvae collected using ovitrap/larval sampling that were reared to adult in the laboratory; F – wild/field-caught female adults sampled in selected sites using sweep net; M – wild/field-caught male adults sampled in selected sites using sweep net. Viral purification: 1- semi-pure samples; 2 – ultra-pure samples. Letters indicate replicate set-ups.

**Additional file 3: Table S14.** BLAST result of the longest CFAV-like contig detected from *Aedes aegypti.*

| Parameter | Results |
| --- | --- |
| evalue | 0 |
| query_start | 42 |
| query_end | 777 |
| hit_start | 6837 |
| hit_end | 7572 |
| align_len | 736 |
| percent_ID | 97.55 |
| query_cov | 94.72 |
| frame | 1 |
| query_seq | CTCTGCAGATATCTTCTTCATAACAGTGTTGTCAACAAAATTCCTGTACTGGTTCCAGGAGAACTGGACTGCACGAATGTACGCCATGAAACACCCGGAGATGGTTAGCTCGATTGGTGGATTCAGATTTGATGAGATTCCCTTCAGGGCTGTCCTTCCCTCTGGTTTTGCCATTGTCGCGATTGCTAGCCTCTCAAGTGTGGTGGTAGGGCTTCTGGCGGCTGGTGTGTTCATGGCCATCATGTACTGCCAGAACAAGTGGAATGCCACCCCGAAGATCTTGACAGCATTGGACGCTCGCGACCAGAGACATGATCGCCCCACAGAAATCACTAGTAGAGTGCCACTCGAAAACACTCGGTCCATCATGTATGCCTTTTGCCTGGTCTTCAGCCTGTTTTGGGCTTTCTGCACACGGTCTCCTGGAGACTTTCTCCGAGGGTCATTGGTGGTCGGGGCTAGCATGTGGCAAATTCTCCATCCCCGGTCAAAGATCCATGATGTCATGGATTTTGGCTCCATGGTGTCTGCCATAGGGTTGCTAGAAATGAACTACCTTTTTTATAGGTTCATGCACATTGCCGCTAGAGCCTTGGGGGCGGTAGCCCCCTTCAACCAGTTTCGAGCTCTGGAAAAGTCAACAACGATCGGTCTGGGAATGAAATGGAAGATGACTCTGAATGCTCTTGATGGAGATGCATTCACAAAATACAAATCACGCGGGGTAAATGAGAC |
